# Highly Stable Self-Cleaning Paints Based on Waste-Valorized PNC-Doped TiO_2_ Nanoparticles

**DOI:** 10.1021/acscatal.3c06203

**Published:** 2024-03-15

**Authors:** Qaisar Maqbool, Orlando Favoni, Thomas Wicht, Niusha Lasemi, Simona Sabbatini, Michael Stöger-Pollach, Maria Letizia Ruello, Francesca Tittarelli, Günther Rupprechter

**Affiliations:** † Department of Materials, Environmental Sciences and Urban Planning (SIMAU), Università Politecnica delle Marche, INSTM Research Unit, via Brecce Bianche 12, 60131 Ancona, Italy; ‡ Institute of Materials Chemistry, TU Wien, Getreidemarkt 9/BC, A-1060 Vienna, Austria; § University Service Center for Transmission Electron Microscopy, TU Wien, 1040 Vienna, Austria

**Keywords:** green synthesis, waste upcycling, photocatalysis, self-cleaning paints, doped TiO_2_ nanoparticles

## Abstract

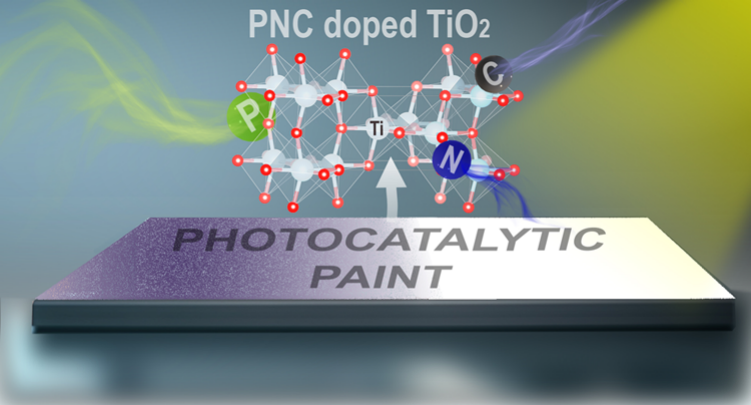

Adding photocatalytically active TiO_2_ nanoparticles (NPs) to polymeric paints is a feasible route toward self-cleaning coatings. While paint modification by TiO_2_-NPs may improve photoactivity, it may also cause polymer degradation and release of toxic volatile organic compounds. To counterbalance adverse effects, a synthesis method for nonmetal (P, N, and C)-doped TiO_2_-NPs is introduced, based purely on waste valorization. PNC-doped TiO_2_-NP characterization by vibrational and photoelectron spectroscopy, electron microscopy, diffraction, and thermal analysis suggests that TiO_2_-NPs were modified with phosphate (P=O), imine species (R=N-R), and carbon, which also hindered the anatase/rutile phase transformation, even upon 700 °C calcination. When added to water-based paints, PNC-doped TiO_2_-NPs achieved 96% removal of surface-adsorbed pollutants under natural sunlight or UV, paralleled by stability of the paint formulation, as confirmed by micro-Fourier transform infrared (FTIR) surface analysis. The origin of the photoinduced self-cleaning properties was rationalized by three-dimensional (3D) and synchronous photoluminescence spectroscopy, indicating that the dopants led to 7.3 times stronger inhibition of photoinduced e^–^/h^+^ recombination when compared to a benchmark P25 photocatalyst.

In times of environmental pollution, the removal of harmful chemicals becomes increasingly important even in our homes. Self-cleaning photocatalytically active wall paints that operate in natural light are particularly promising. Considering that most of the exposed interior or exterior surfaces of buildings are coated with paints, there is a huge interest in improving paint properties by modifying polymeric paints with photocatalysts such as TiO_2_ nanoparticles (NPs).^1−4^ Adding TiO_2_-NPs to water-based acrylic paint induces photocatalytic activity but also reduces the chemical stability of the paint polymer matrix by degradation. Even worse, due to photocatalysis, the paint matrix may release hazardous volatile organic compounds (VOCs), which can cause serious human health issues.^4,5^ Overcoming the drawback of photocatalytic polymer paint degradation, while maintaining the self-cleaning properties, requires a targeted modification of TiO_2_-NPs, which so far rarely resulted in stable TiO_2_-NP-based paint formulations. One route is adding nonmetal dopants, like carbon nanotubes (CNTs),^4^ but in light of the high cost and low biocompatibility of CNTs,^6^ such modifications are unlikely to affect the global paint market.

Most importantly, the photocatalytic performance of semiconductors such as TiO_2_-NPs depends on the photoinduced band gap excitation (band gap energy), structural defects, generation of electron–hole pairs (e^–^/h^+^), inhibition of (e^–^/h^+^) recombination, and successful charge transfer toward the surface for radical formation.^7,8^ Unfortunately, TiO_2_-NPs (anatase/rutile) possess a wide band gap (3–3.2 eV) and poor tendency of (e^–^/h^+^) separation. This requires modification of TiO_2_-NPs through metal ion doping,^9,10^ nonmetal ion doping,^11,12^ or codoping.^13−15^ Various modification methods have been adopted for TiO_2_-NPs, such as N-doping through pulsed laser deposition,^16^ Pt or B doping, N and C, Fe, N, S, Pr, and P codoping through sol–gel methods,^13,17−19^ Sr and N doping by hydrothermal methods,^20,21^ Pd or Au doping by photodeposition,^22^ Ag and SnO_2_ codoping by photoreduction,^23^ etc. However, frequently adopted methods (e.g., sol–gel synthesis) for TiO_2_-NP modification often utilize synthetic reagents as precursors mined from raw materials, thus making the process unsustainable. As the annual consumption of natural resources is faster than their replenishment,^24−26^ sustainable nanosynthesis (SNS) is difficult to achieve. Preservation of natural resources, nanomaterials (NMs) for circular economy, clean synthesis methods, and waste valorization to NMs are important but under-represented areas in nanoscience and technology.

Green chemistry may provide a route toward achieving SNS. However, carefully evaluating green chemistry principles (GCP),^27^ UN sustainable development goals (SDGs), and the US individual waste reduction model (iWARM) of the Environmental Protection Agency (EPA),^28,29^ the process of NM synthesis via existing methods cannot be truly classified as “green”. It requires an entirely different approach, a method that can valorize potential waste as a starting raw material, being minimally invasive to the environment by utilizing byproducts of the NM nanosynthesis process and at the same time being scalable to an industrial level.^30^

Our work presented herein demonstrates an end-to-end methodology, describing for the first time SNS and an application-based performance of the obtained materials. First, a novel method of simultaneous waste (organic plus inorganic) valorization to nonmetal (P, N, and C)-doped TiO_2_-NPs is elaborated, paralleled by mechanistic studies of the SNS process by attenuated total reflectance–Fourier transform infrared spectroscopy (ATR-FTIR). The PNC-doped TiO_2_-NPs were thoroughly characterized by diffuse reflectance infrared Fourier transform spectroscopy (DRIFTS), micro-Raman, X-ray diffraction (XRD), high-resolution transmission electron microscopy (HR-TEM), energy-filtered transmission electron microscopy coupled with electron energy loss spectrometry (EFTEM-EELS), selected area electron diffraction (SAED), X-ray photoelectron spectroscopy (XPS), and thermogravimetry (TG). Second, the photocatalytic potential in terms of photoinduced electron–hole (e^–^/h^+^) pair separation of PNC-doped TiO_2_-NPs was studied by three-dimensional synchronous photoluminescence (3D-SPL) spectroscopy. Third, the obtained PNC-doped TiO_2_-NPs were successfully used as a stable photocatalytic additive to water-based paints. The excellent self-cleaning performance of the modified paints was investigated through customized online UV–vis spectroscopy and natural sunlight photocatalysis. At last, the effect of PNC-doped TiO_2_-NPs on paint stability was analyzed by micro-FTIR spectroscopy. Overall, PNC-doped TiO_2_-NPs demonstrated 96% removal of surface-adsorbed pollutants with excellent stability in paint formulations.

## Results and Discussion

### Sustainable Nanosynthesis: Characterization of Materials and Mechanistic Study

**Figure 1:**
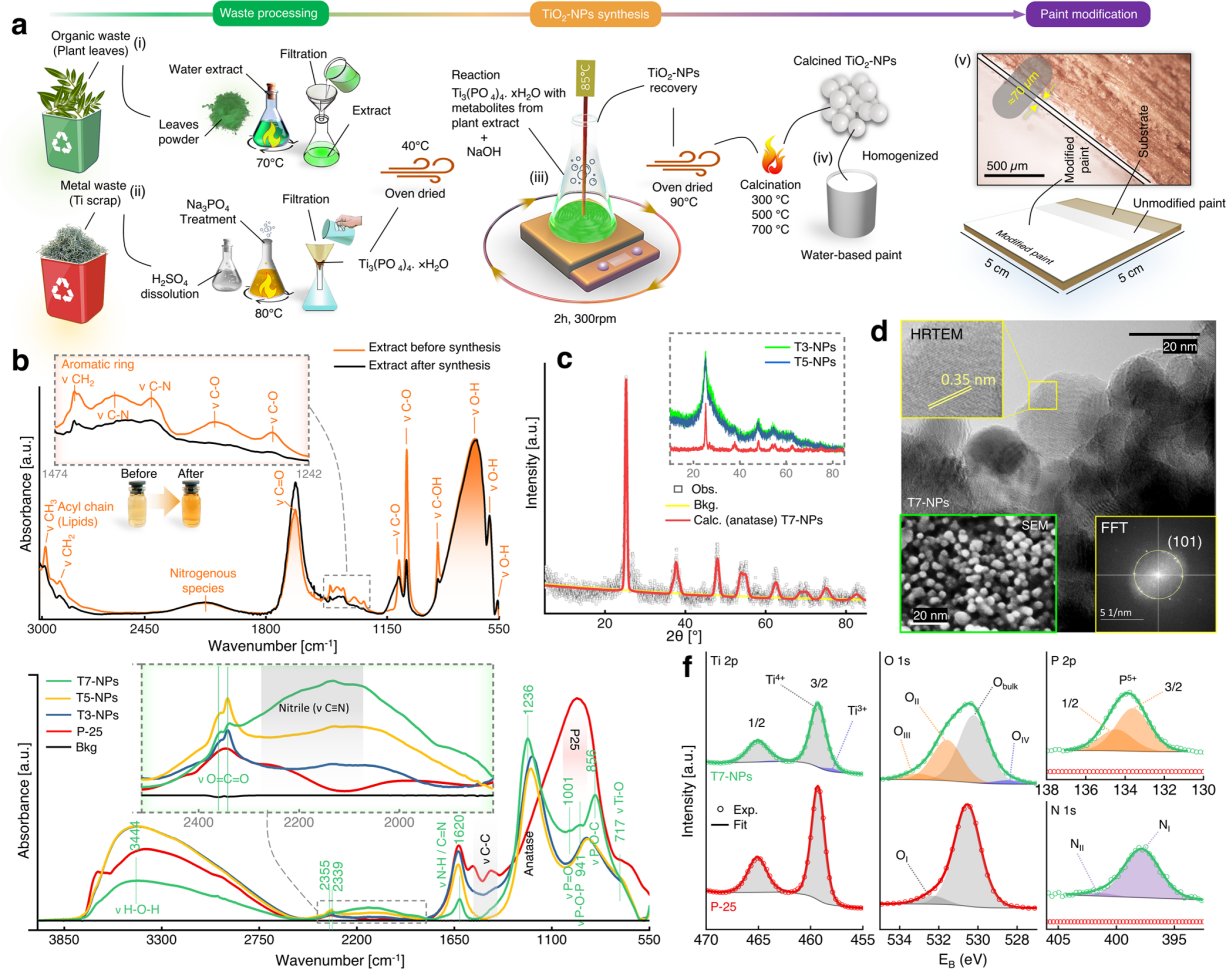
Sustainable nanosynthesis and characterization of prepared photocatalysts. (a) Schematics elaborating the green and sustainable nanosynthesis (SNS) of phosphate, nitrogen, and carbon (PNC)-doped titanium oxide nanoparticles (TiO_2_-NPs) via metal and organic waste valorization, their use as a photocatalytic additive to water-based paint, and optical micrograph showing a homogeneous modified paint as a surface coating over the substrate (wood). (b) ATR-FTIR spectra of the organic waste (fallen leaves) extract before starting the synthesis reaction (orange) and after the recovery of TiO_2_-NPs (black): inset: magnified region (1242–1474 cm^–1^). (c) Crystal structure of TiO_2_-NPs (calcined at 700 °C labeled as T7-NPs) via Rietveld refinement. Inset: measured diffractograms of the as-synthesized TiO_2_-NPs (calcined at 300 °C: T3-NPs and at 500 °C: T5-NPs). (d) HRTEM of unsupported T7-NPs with magnified lattice spacing as an inset (top left), FFT (bottom right), and SEM of T7-NPs (bottom left) showing homogeneous anatase-TiO_2_-NPs. (e) DRIFTS spectra of T3-NPs (blue), T5-NPs (orange), T7-NPs (green), and P25 (red). Inset: magnified region (1800–2500 cm^–1^). (f) XPS spectra of T7-NPs (green) and P25 (red).

SNS is illustrated in Figure 1a, employing naturally occurring secondary metabolites from organic waste to reduce waste-derived Ti_3_(PO_4_)_4_·*x*H_2_O to X-doped TiO_2_-NPs (X = P, N, and C). The efficacy of the presented SNS methodology represents substantial progress in the field of classical green chemistry methods,^31−39^ by abolishing the use of fresh plant resources. Living plants can produce oxygen via the photosynthetic route to balance the ecosystem instead of being used for the extraction of secondary metabolites. In addition, synthetic metal salts are not used as primary precursors for the synthesis of NPs. Instead, the current approach enables the utilization of residual plant biomass (organic waste) and industrial metal scrap (such as Ti), which are conventionally regarded as inorganic waste, as viable alternatives to synthetic reagents. While metal waste is typically recycled to produce materials of the same class, the utilization of low-cost metal waste to produce high-value, functionalized materials (e.g., nonmetal doped NPs) has not yet been fully acknowledged. Despite using sulfuric acid and high-temperature annealing, the SNS method still adheres to most green chemistry principles and supports the UN SDGs through heterogeneous waste reduction and natural resource conservation. Therefore, the SNS method presented in this research represents a significant advancement in the green synthesis of NPs.

To evaluate the mechanism of the synthesis of X-doped TiO_2_-NPs, liquid-phase ATR-FTIR analysis of the organic waste extract was carried out before and after synthesis, as shown in Figure 1b. Comparative analysis clearly shows changes in four regions: (i) A change in intensity at 878 cm^–1^, characterizing νC–OH of the H-bond (OH torsion), typical of flavonoids;^40^ interestingly, the Ti_3_(PO_4_)_4_·*x*H_2_O reaction with the metabolic extract showed more affinity to flavonoids than polyphenolic compounds during the redox process of metal ion chelation and reduction to NPs.^41^ (ii) A sharp decline in peaks at 1044, 1085, and 1274 cm^–1^, suggesting reduction of νC–O bands linked to carboxylic acid, ether, alcoholic groups, and νC–O stretching of carbonyl groups, respectively;^42^ in the same region, peak shifts of 1044 → 1045 cm^–1^ and 1085 → 1087 cm^–1^ further confirm consumption of metabolite mass, as the vibrational frequency is inversely proportional to the oscillator mass.^43^ (iii) Most importantly, utilization of nitrogenous metabolites (νC–N stretching vibrations related to aromatic amines and alkaloids at 1326–1419 cm^–1^^42^) (inset; Figure 1b) may have facilitated the formation of small size X-doped TiO_2_-NPs, consistent with the literature.^44−46^ (iv) Equally important is the utilization of flavonoids, showing aromatic ring stretching (νCH_2_) at 1456 cm^–1^^47^ and lipids, which is evident from their acyl CH_2_/CH_3_ stretching vibrations at 2903–2980 cm^–1^.^48^ It is possible that together with nitrogenous compounds, utilization of lipids may act as surface-stabilizing agents for achieving homogeneous and small size X-doped TiO_2_-NPs, also reported previously.^49^ The remaining two unaffected regions, i.e., 558–840 and 1900–2300 cm^–1^, likely correspond to polyphenolic or nitrogenous compounds.^50−52^ Overall, the ATR-FTIR study of the SNS process revealed involvement of nitrogen- and carbon-related species during the reaction of Ti_3_(PO_4_)_4_·*x*H_2_O with the extract. Thus, the synthesized materials should exhibit P, N, and C doping, which is further examined below.

The crystallography of as-synthesized T3-, T5-, and T7-NPs (calcined at 300, 500, and 700 °C, respectively) was examined by XRD. The diffraction patterns of T7-NPs (Figure 1c) and T3- and T5-NPs (inset: Figure 1c) revealed the effect of different thermal treatments. The broad peaks in T3-NPs/T5-NPs are in good agreement with COD 1526931 (see Figure S1 for the crystal structure Rietveld refinement of T3- and T5-NPs), suggesting small crystallite size (even up to 500 °C), as compared to sharp peaks of T7-NPs (at 700 °C). Although T7-NPs show a good fit to anatase (COD 1526931), when magnified, the effect of nonmetal dopants can be observed (Figure S1). No phase transformation was detected, even after 700 °C calcination, which further indicates that the nonmetal dopants hinder the anatase/rutile phase transformation.^53−55^ The crystallite size of all photocatalysts was calculated using Debye–Scherrer’s equation (see eq S4), with average crystallite sizes of 3.38, 2.10, 9.62, and 14.32 nm for T3-, T5-, and T7-NPs and P25, respectively. Crystallographic parameters are detailed in Table S1.

The crystal structure was further confirmed by HRTEM and selected area electron diffraction (SAED). HRTEM showed lattice fringes with a spacing of 0.35 nm (Figure 1d), corresponding to anatase-phase titania (101). The FFT of the HRTEM image also confirmed the lattice spacing of titania. The SAED pattern also showed diffraction spots associated with anatase,^56^ as shown in Figure S2 and Supporting Table S2. Moreover, oval shape T5-NPs of ≈20 nm are displayed in Figure S2.

After SNS, DRIFTS analysis (Figure 1e) was used to trace the phosphorus, nitrogen, and carbon species interacting with the anatase TiO_2_ matrix. DRIFTS can detect even small amounts of surface species, adsorbed molecules, or even dopants producing relatively strong and detectable spectral features.^57,58^

As expected, distinctive νP–O–C and νP–O–P deformation vibrations and νP=O stretching bands were clearly visible in the region of 850–1000 cm^–1^ for T3-, T5-, and T7-NPs. In particular, an increased peak intensity was recorded after calcination at 700 °C (T7-NPs).^59−61^ Moreover, the broad region (1000 cm^–1^) in T7-NPs further suggests that there might be the presence of −Ti–O–N–Ti– and N–Ti–O bond vibrations, as reported previously.^62^ Nitrogen species were traced at around 1580 to 1750 cm^–1^, mainly νN–H^63^ and νC=N (imine group).^64^ The appearance of a broad peak around 2200 cm^–1^ indicates that above 500 °C, imines were dehydrated to form nitriles, which further suggests the coexistence of nitrogen species in different ratios. The existing nitrogen-containing functional groups may play a critical role in the generation of surface oxygen defects. Overall, DRIFTS revealed the dynamic nature of the functional groups on the surface of T3-, T5-, and T7-NPs, with the thermal treatment probably resulting in the peak shifts.^65−67^

A broad peak, signature of P25 (representing anatase and rutile), can been seen at 700–1350 cm^–1^,^68^ further overlapping with the νC–C region (1350–1580 cm^–1^), probably from amorphous carbon.^41^ Similarly, the broad peak related to anatase, centered at 1236 cm^–1^, was prominent for T3-, T5-, and T7-NPs. Moreover, νH–O–H (stretching vibrations) was traced by a broad peak, centered at 3444 cm^–1^. In summary, the surface chemistry analysis by DRIFTS confirmed that X = P, N, and C was present on the surface of anatase TiO_2_-NPs, thus labeled as PNC-doped TiO_2_-NPs.

The nonmetal dopants (e.g., phosphate, nitrogen, and carbon) traced by DRIFTS were further characterized by XPS. Figure 1f shows the XPS Ti 2p, O 1s, P 2p, and N 1s spectra of P25 and T7-NPs. XPS spectra of T3- and T5-NPs are shown in Figure S3 and were quite similar to T7-NPs. The measured Ti 2p peak shape, doublet separation (DS) of 5.8 eV,^69^ and α′ of ∼873^70^ agree well with the literature data of TiO_2_. The peak areas indicate a Ti amount of 22.5 atom % in P25 and 13.2 atom % in T3-NPs. Furthermore, for the modified NPs, there appears to be a second Ti species at around 1.3 eV lower energy, making up around 5% of the total Ti amount and likely corresponding to Ti^3+^ and/or Ti^2+^ due to oxygen vacancies.^71,72^ The O 1s main peaks agree with the literature value of 530.4 eV^73^ (P25: 530.4 eV, T7-NPs: 530.3 eV) and a ΔTi 2p O 1s of 71.1 eV^73^ (P25: 71.2 eV, T7-NPs: 70.9 eV) for oxygen in bulk TiO_2_. For P25, a small peak shoulder O_I_ at 532.3 eV originates from surface hydroxyl groups (Ti–OH).^74^ In T7-NPs, shoulders O_II_ and O_III_ at a BE of 531.6 and 533.1 correspond to P=O and P–O–P from (di)phosphate, respectively.^75^ Additionally, hydroxylic groups as well as O–Ti^3+^ (531.2 eV)^71,72^ may contribute to O_II_. Lastly, O_IV_ at 528.3 eV aligns with O–Ti^2+^.^71,72^ The total amounts of oxygen are 56.4 atom % for P25 and 57.0 atom % for T7-NPs.

For the T3-, T5-, and T7-NPs, the P 2p region showed a broad peak corresponding to the doublet of phosphorus with a DS of 0.86 and a 2p_3/2_ BE of ∼133.6 eV, corresponding to P^5+^ in phosphate (PO_4_)^3–^ and/or diphosphate (P_2_O_7_)^4–^.^75−78^ In agreement with DRIFTS, the highest amount of phosphate (10.0%) was detected for T7-NPs.

The N 1s region revealed 5.4% nitrogen for T7-NPs, via a broad peak with a maximum at ∼398 eV (N_I_). Additionally, a shoulder at ∼401.5 eV (N_II_) was more pronounced for T3- and T5-NPs. XPS identification of organic nitrogen species is neither trivial nor is there a clear consensus in the literature. Nonetheless, the BE maximum at ∼398 eV is in best accordance with imine, nitrile, or pyridinic species, matching well with DRIFTS. The shoulder at higher BE may originate from graphitic nitrogen or protonated/hydrogenated nitrogen species.^79−81^

C 1s spectra are shown in Figure S3 and discussed in Supporting Text S1. Moreover, the elemental percentage of each specimen is given in Supporting Table S3. Overall, the XPS data are well in line with and corroborate the FTIR, XRD, and electron microscopy results.

**Figure 2:**
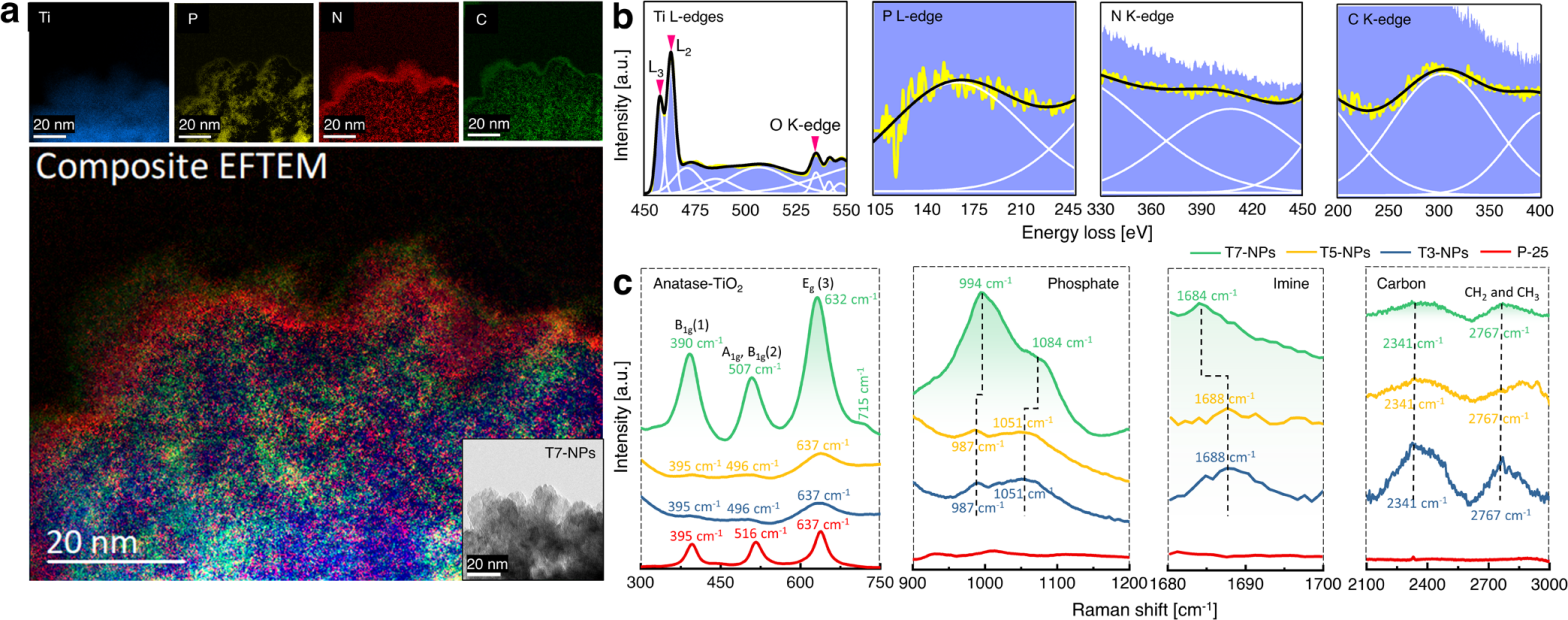
Analysis of T7-NPs (PNC-doped TiO_2_ calcined at 700 °C) by EFTEM and Raman spectroscopy. (a) Energy-filtered transmission electron microscopy maps of Ti, P, N, and C of agglomerated PNC-doped nanoparticles (T7-NP). (b) EELS spectra corresponded to Ti L_3,2_-edges, P L-edge, N K-edge, and C K-edge. Peak smoothing was performed using the Savitzky–Golay method with a window size of 9 and a polynomial order of 2. Cumulative Gauss fits (solid white line) for EELS spectra and corresponding deconvoluted peaks are selected based on the best *R*^2^. (c) Confocal micro-Raman spectroscopy analysis of T7-, T5-, T3-, and P25-NPs.

To further support the DRIFTS and XPS findings and to gain insights into the PNC-modified local structures of TiO_2_ NPs, several microscopic and spectroscopic techniques, including EFTEM/EELS, HRTEM/SAED, and μ-Raman spectroscopy, were applied. The T7-NP sample was selected for further studies by EFTEM and EELS. Color-coded elemental mapping by EFTEM (Figure 2a) together with EELS successfully confirmed the doping of titania with PNC. The visible enrichment of phosphorus and nitrogen at the surface of the NPs rationalizes the high amount of these elements measured by XPS. EELS spectra related to Ti L-edges including L_3_ and L_2_ (457, 462 eV), O K-edge (∼534 eV), P L-edge (∼162 eV), N K-edge (∼407 eV), and C K-edge (∼303 eV) are presented in Figure 2b.

Confocal μ-Raman spectroscopy was carried out by point-mapping five randomly selected areas on each of the photocatalysts (Figure 2c). In agreement with XRD (Figure 1c), μ-Raman also indicated that for all modified NPs, TiO_2_ was in the anatase phase. There are six major Raman active modes for anatase, i.e., A_1g_ + 2B_1g_ + 3E_g_.^82^

The E_g_(1) peak of P25 was detected at 144 cm^–1^ (Figure S4). For T3-, T5-, and T7-NPs, it displayed a red shift and peak broadening and appeared at ∼150 cm^–1^ due to the smaller NP sizes as compared to P25. Peak broadening as a result of phonon confinement was more significant for T3- and T5-NPs, which is consistent with XRD crystallite size data, as their sizes (size <10 nm) were much smaller than those of P25 and T7-NPs.

For the E_g_(2) vibration (Figure S4), a low-intensity Raman active peak was detected around 196 and 200 cm^–1^ for P25 and T7-NPs, respectively, overlapping with E_g_(1) for the smaller crystallite sizes of T3- and T5-NPs.

The B_1g_(1) and E_g_(3) vibrations of T7-NPs showed a larger red shift than for P25 and T3- and T5-NPs and they were detected at 390 and 632 cm^–1^, correspondingly, while A_1g_ and B_1g_(2) of T3- and T5-NPs appeared at lower wavenumber (∼496 cm^–1^) than those of P25 and T7-NPs. However, T7-NPs also showed a red shift with respect to P25, possibly due to the interaction of dopants (P, N, or C).

Furthermore, Raman peaks of phosphate, imine, and carbon were also identified, complementing the XPS and DRIFTS analysis. A weak diphosphate P–O–P vibration (Figure 2c) at 715 cm^–1 ^^83^ was detected for T7-NPs. This vibration peak could not be detected for the small crystallite size T3- and T5-NPs due to peak broadening. Deconvoluted peaks related to phosphate vibrations of P=O stretching^84^ were red-shifted for T3-/T5-NPs when compared to T7-NPs, showing a broad double peak (987, 1051 cm^–1^ and 994, 1084 cm^–1^, correspondingly).

The imine group^85^ was detected at 1688 cm^–1^ for T3- and T5-NPs, while for T7-NPs, it was slightly red-shifted to 1684 cm^–1^. Additionally, peak broadening and a red shift in Raman active modes of titania are not only due to phonon confinement but also occur due to doping with nitrogen, as reported in the literature.^86^

Raman spectroscopy revealed mainly amorphous carbon combined with disordered graphite. A broad amorphous carbon peak was detected for T3- and T5-NPs at 1460 cm^–1^ (Figure S5). The G (graphite) band and D (defect) band showed a broad doublet for T7-NPs (1405, 1563 cm^–1^). Graphitization also slightly increased by increasing the calcination temperature. A broad peak of amorphous carbon at 2341 cm^–1^ with C–C vibration at 2767 cm^–1^ was also detected by Raman. No carbonization was evident for P25.

Complementing DRIFTS, XPS, and Raman analysis, commercial P25 as a reference and the prepared T3-, T5-, and T7-NPs were further analyzed through thermogravimetry (TG), derivative thermogravimetry (DTG), and simultaneous differential thermal analysis (SDTA), as shown in Figure S6 and Supporting Table S4, to assess the crystalline stability. For each specimen, the main temperature intervals (Δ*T*n) with the corresponding weight loss and temperatures of maximum decomposition (Tn), if observed, are reported in Supporting Table S4.

Comparing the thermal behavior of T3-, T5-, and T7-NPs with commercial P25, the synthesized NMs, even after calcination, also showed a weight loss from 35 to 125 °C ascribed to removal of water adsorbed upon exposure to ambient after calcination, promoted by the high specific surface of the NMs. However, for the SNS materials, the total weight loss was significantly higher than for P25 (1.4%), about 18% (T3-NPs), 15% (T5-NPs), and 4% (T7-NPs), whether or not in N_2_ or air. This is due to the higher content of carbon-based compounds in the manufactured NMs, which decompose below 750 °C. Indeed, the total weight loss decreases with the increase of calcination temperature; moreover, as expected, only for specimens calcined at *T* = 300 and 500 °C, a peak of maximum decomposition (Tn) at 600 °C appeared. At higher temperatures, with a Tn at 900 °C, also, phosphorus–oxygen compounds decomposed, explaining the continued weight loss up to 1300 °C, as reported previously.^87^

In N_2_, the further weight loss at high temperatures may also be assigned to high-temperature TiO_*x*_ reduction into TiC due to pyrolyzed carbon, as reported previously.^41^ In all prepared PNC-doped TiO_2_-NPs, the endothermal transition of TiO_2_-NPs from anatase to rutile at *T* > 500 °C^88−90^ was not observed. This perfectly matches the crystallographic (XRD) and morphological (HRTEM) studies, which did not detect a phase transformation, even upon 700 °C calcination, which once more indicates that the nonmetal dopants suppress the phase transformation. The TG, DTG, and SDTA behavior of all photocatalysts is further detailed in Supporting Text S2.

### Photoinduced Electron–Hole (e^–^/h^+^) Pair Separation Studied by 3D and Synchronous Photoluminescence (SPL) Spectroscopy

**Figure 3:**
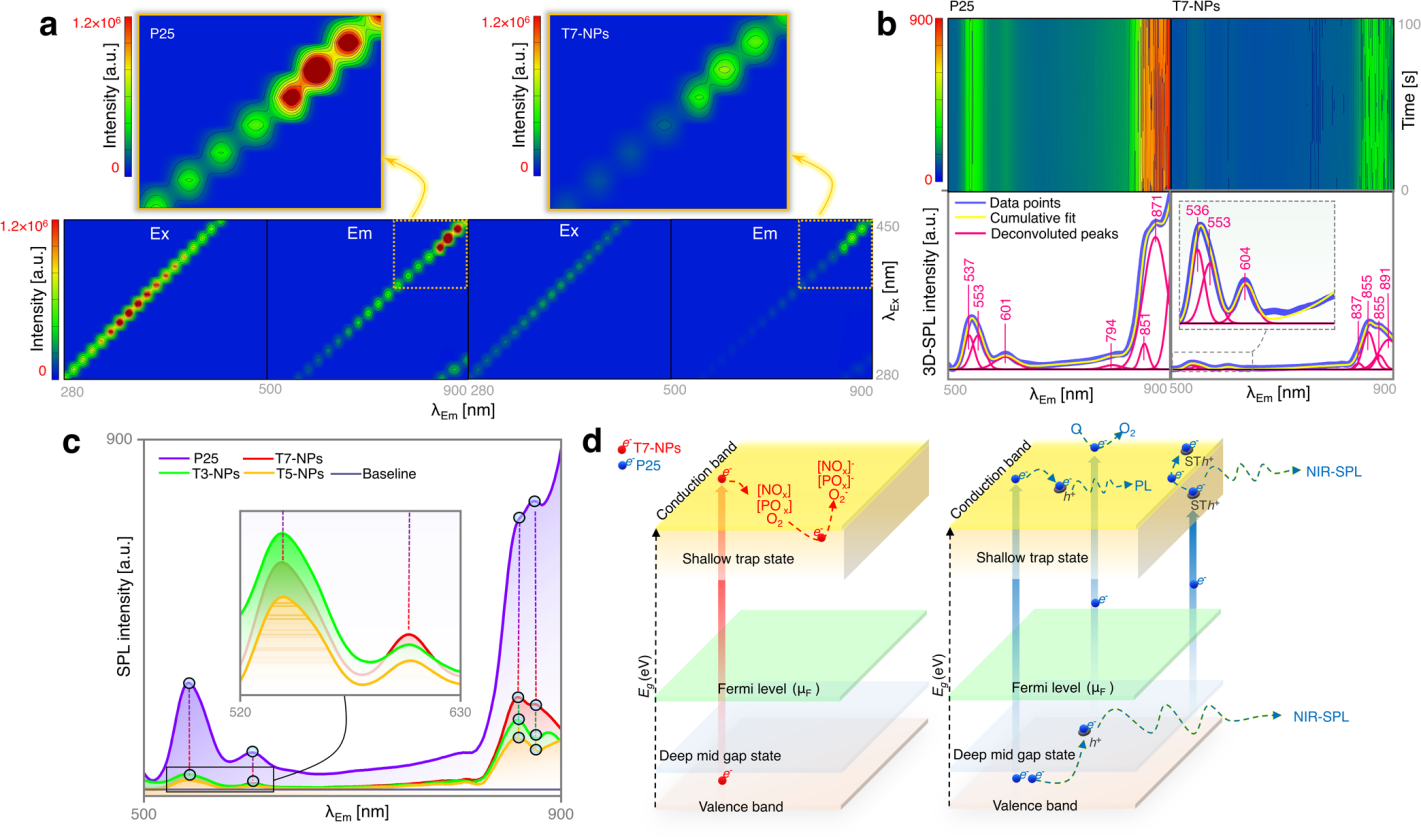
Photoinduced electron–hole (e^–^/h^+^) recombination analysis in PNC-doped TiO_2_-NPs. (a) 3D photoluminescence (PL) spectroscopy, showing excitation–emission spectrum contour plots of P25 and T7-NPs (for 3D PL spectra of T3- and T5-NPs, see Figure S7). (b) Synchronous PL (SPL) spectroscopy of P25 and T7-NPs with a fixed wavelength difference of 200 nm (Δλ = λ_Em_- λ_Ex_ = 200 nm), λ_Ex_ starting from 300 nm and λ_Em_ from 500–900 nm. SPL spectra of T3-NPs and T5-NPs are shown in Figure S7. (c) Comparison of SPL of P25, T3-, T5-, and T7-NPs at the same (*y*-axis) intensities. (d) Mechanism of PL, based on results shown in (a) and (b).

The photocatalytic performance of TiO_2_-NPs strongly depends on their ability to resist the recombination of photoinduced electron–hole (e^–^/h^+^) pairs, typically at surface defects and impurities, which can be evaluated by photoluminescence (PL) spectroscopy. The faster the e^–^/h^+^ recombination, the higher the PL intensity is, yielding a poorly active photocatalytic surface. Although TiO_2_ has a band gap in the UV region with decreased or no PL emission in the visible range, some recent studies showed that defects (e.g., oxygen vacancies or structural defects) can cause emission in the visible or near-infrared (NIR) regions.^7,11,91−94^ Considering this, 3D PL spectroscopy (excitation–emission spectrum contour plots) can simultaneously measure the band gap luminescence intensity as a function of excitation wavelength (λ_Ex_) and emission wavelength (λ_Em_) changes, as shown in Figure 3a. It is obvious that P25 has high PL intensity when compared to T7-NPs, and PL λ_Em_ spots are concentric only in the visible region with even high intensities toward the NIR range. The 3D PL intensity of T3- and T5-NPs is displayed in Figure S7.

Compositional defects due to PNC doping and surface defects due to oxygen vacancies and Ti^3+^ in TiO_2_-NPs can be further evaluated through synchronous PL (SPL) spectroscopy. Figure 3b shows the SPL spectra with a fixed wavelength difference of 200 nm (Δλ = λ_Em_ – λ_Ex_ = 200 nm), λ_Ex_ starting from 300 nm and λ_Em_ from 500–900 nm. Considering that the λ_Ex_ energies also include band gap energies of the materials, it is most likely that absorption should overall affect the e^–^ transition from the valence band (VB) to the conduction band (CB) as well as interference with shallow trap states (STS) beneath the CB, connected with oxygen vacancies (OV), surface oxygen vacancies (SOV), structural defects (SD), impurities, and unbalanced Ti atoms on the surface. The SPL spectrum of P25 can be deconvoluted (using Gaussian function) into six different peaks, centered at 537, 553, 601, 794, 851, and 871 nm, and an overlapping NIR region (exceeding the observation window, i.e., 900 nm). The SPL peaks at 537 and 553 nm (green region) arise from the recombination of e^–^ with h^+^ in STS, the h^+^ that originated from intrinsic point defects, such as OV, typical of the n-type nature of TiO_2_-NPs.^91^ Interestingly, Figures 3c and S7 reveal that the SPL peak in this region is 7.3 times more prominent for P25 than for the other materials (e.g., T7-NPs), suggesting poor charge (e^–^) relaxation in P25 toward STS near the CB. The SPL peak at 601 nm (orange-red region), which was quite sharp for P25, was broader and less intense for the other samples due to the presence of uncoordinated Ti^3+^ and recombination of e^–^/h^+^ in the VB. The SPL peak at 780–790 nm (red region), relatively small in P25 but undetectable in T7-NPs, may have possibly emerged due to SOV.

The SPL spectra in the NIR region (NIR-PL) further explain recombination or separation of photoinduced electron–hole (e^–^/h^+^) pairs in all photocatalysts. NIR-PL spectra (Figure 3b,c) suggest that the λ_Em_ emission can happen in the NIR-PL region only if the photon energy surpasses the band gap energy of TiO_2_. This means that this necessarily involves recombination of e^–^/h^+^ between VB, STS, and CB. In the O_2_-rich environment around the surface, particularly when SPL was performed in air, e^–^ should be scavenged by surface O_2_ and one expects to observe O_2_-induced PL quenching. Interestingly, there was robust depletion of e^–^ by O_2_, as T3-, T5-, and T7-NPs have shown almost 5.5, 6.8, and 3.9 times lower NIR-PL intensity than that of P25, respectively. Perhaps the involvement of dopants (phosphates P=O or P–O–P), imine or nitrile species (R=N-R), and possibly C species, which is evident from XPS analysis, may have aided in the successful separation of photoinduced e^–^/h^+^ in CB and STS,^93^ as illustrated in Figure 3d. It is important to point out that NIR-PL quenching or enhancement depends on the nature of dopants (metallic vs nonmetallic). Most likely, introduction of metal as a dopant (e.g., vanadium) can enhance NIR-PL in TiO_2_ due to the Jahn–Teller effect.^94^ Contrary to that, in our case, the presence of an optimum carbon content along with P and N species acted as strong inhibitors of e^–^/h^+^ recombination.

On the other hand, the contradicting performance of P25 vs T7-NPs, i.e., the enhanced NIR-PL in the presence of O_2_, was observed before. Since P25 includes the rutile phase as well, there are two hypotheses, which may explain this phenomenon: (i) radiative recombination of trapped h^+^ with free e^–^ or (ii) radiative recombination of trapped e^–^ with free h^+^.^7^ For the first case, there are studies which suggested that photoinduced free h^+^ at the surface can bind to O atoms of rutile (100) or (110) surfaces and produce self-trapped h^+^ (STh^+^),^95,96^ which can then combine with e^–^ in CB to generate NIR-PL or other reactions responsible for oxygen photoevolution.^97^ The formation of STh^+^ may also occur in the subsurface region of STS, which can then recombine with photogenerated e^–^, leading to NIR-PL. In such a case, it is possible that exposure of the rutile surface to O_2_ produces superoxide O_2_^–^, which can result in upward band bending in the TiO_2_ surface and aggregation of STh^+^ to the STS. This suggests that O_2_ can act as an e^–^ scavenger as well as facilitate STh^+^ formation to the STS, which can result in e^–^/STHh^+^ recombination with NIR-PL. On average, the e^–^/STh^+^ recombination dominates over e^–^ scavenging by O_2_ in the CB.

As NIR-PL in P25 suggests that e^–^ are not sufficiently scavenged by surface O_2_, the second hypothesis (i.e., radiative recombination of trapped e^–^ with free valence band h^+^) most likely applies. The involvement of deep midgap states, below the Fermi level (μ_F_), may support this hypothesis. The already occupied region with fair density of free h^+^, even before photoexcitation, can hinder the e^–^ pathway to the CB to combine with O_2_. It is most likely that these free h^+^ in midgap states lead to radiative recombination of e^–^, resulting in intense NIR-PL.^98^

Finally, UV–vis absorbance spectra of all specimens were acquired, shown in Supporting Figure S8. From a Tauc plot, it is obvious that the band gap energy of T7-NPs was significantly reduced to 2.86 eV, as compared to P25 (3.00 eV), possibly due to the phase stability even at high temperature (700 °C), oxygen defects, and most importantly the dopant effect. The improved light absorbance, shifted more toward visible λ (inset of Figure S8), is consistent with the literature.^11,17^ Overall, based on 3D-PL, 3D-SPL, and band gap energy results, the PNC-doped TiO_2_-NPs showed much better photocatalytic performance than that of P25.

### Photocatalytic Performance of Paint Modified by PNC-Doped TiO_2_-NPs

#### Pollutant Quantification by Customized Online UV–Vis Spectroscopy

**Figure 4:**
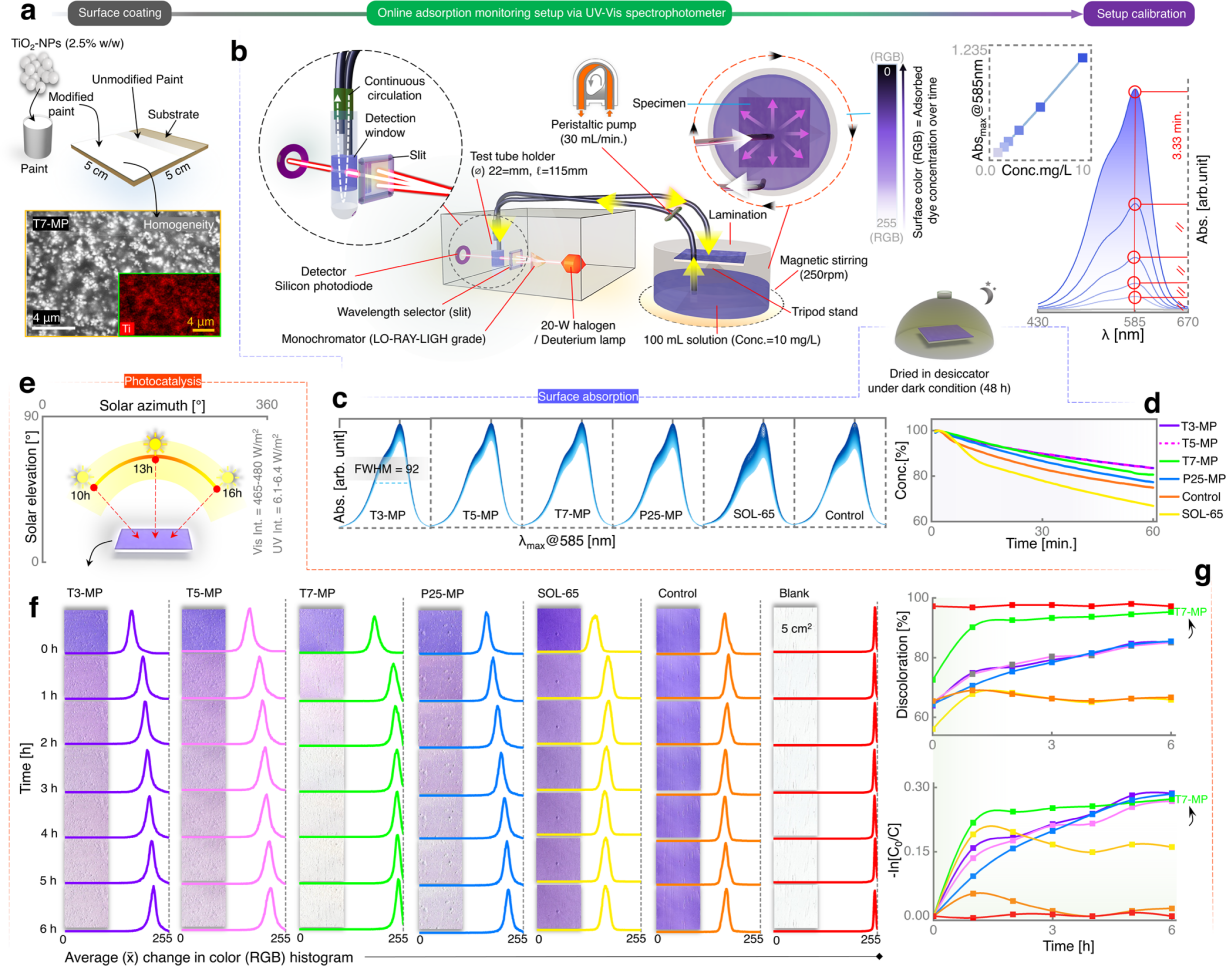
Modification of paint with PNC-doped TiO_2_-NPs, surface adsorption, and photocatalysis. (a) Addition of PNC-doped TiO_2_-NPs to the paint and application to an inert substrate (wooden sheet), called modified paint surface (e.g., T3-MP). SEM showing homogeneity of the TiO_2_-NPs in the paint matrix. (b) Schematics (left) describing a customized UV–vis spectroscopy setup for continuous online monitoring of pollutant adsorption (methyl violet, MV) on the MP surface. Calibration of the setup (right; see more details in the Methods, Supporting Information). (c) Online UV–vis absorption spectra (1 reading/1 min until 1 h, Abs. max.=585 nm) showing changes in MV concentration due to adsorption on T3- and T5- and T7-MP, P25-MP, and SOL-65. MV adsorption over unmodified paint as a “control”, with the unmodified paint surface without any MV adsorption as a “blank”. (d) Change in MV concentration over time as observed in (c). (e) Photocatalytic conditions under natural sunlight, for daytime 10–16 h, and showing the respective solar elevation [°] and solar azimuth [°]. (f) Photocatalytic performance (rate of discoloration) after MV adsorption, drying and exposure to natural sunlight (vis. light int.= 465–480 W/m^2^, UV light int. = 6.1–6.4 W/m^2^) showing discoloration in terms of average (*x̅*) RGB color histogram change (*x̅* RGB magnitude of 255 for white and 0 for black) over time. (g) Discoloration percentage over time [h] and −In of measurement in (f). “Blank” is not a performing sample but a sample without MV exposure, representative of the maximum discoloration value which can be achieved by photocatalysis.

PNC-doped TiO_2_-NPs and P25 were used as additives (2.5% w/w) to modify a water-based paint, subsequently applied over an inert substrate, as shown in Figure 4a. The homogeneity of all PNC-doped TiO_2_-NPs within the paint can be seen in Figure S9. The modified paints (MPs; e.g., T3-MP) were first exposed to surface pollutants (i.e., 100 mL of 10 mg/L solution of methyl violet 2B) (also called Basic Violet 1 and simply termed methyl violet (MV) below) at room temperature (see the Methods section in the Supporting Information). MV adsorption on the MPs was measured using a customized online UV–vis spectroscopy setup (absorption vs time), as shown in Figure 4b (left) and calibrated in Figure 4b (right). Each MP surface was exposed to MV for 1 h under a continuous flow, and changes in MV concentration due to surface adsorption were recorded every minute (1 reading/1 min until 1 h, Abs. max.= 585 nm). It is apparent from Figure 4c,4d that T3- and T5-MPs adsorbed 16%, with T7-MPs adsorbing 20% and P25-MPs adsorbing 22% of MV, with stable adsorption over time until 1 h. The MV adsorption trend remained unaffected for repeated cycles (Figure S10). Interestingly, for SOL-65 (a commercial photocatalytic paint), the first MV exposure resulted in swifter adsorption of 14% in the first 15 min, which became steadier after 15 min and remained constant over the next cycle (Figure S10). In total, SOL-65 adsorbed 33% of MV in 1 h, i.e., twice as much as T3- and T5-MP. After the course of MV adsorption, all MPs were dried in the dark (48 h), prior to further photocatalytic studies.

Adsorption of MV is related to a change in the color intensity of the MP surface. A correlation between MV absorbance and color change was observed (see the Methods in the Supporting Information). The higher the MV absorbance, the more intense the color was and the lower the average RGB color histogram values were (magnitude of 255 for white and 0 for black) and vice versa. Accordingly, the photocatalytic rate over the MPs is directly proportional to the rate of discoloration.

#### Photocatalysis under Natural Sunlight and UV Light

After exposure to MV (Figure 4b) and quantification of adsorbed MV (Figure 4c,d), the specimens were dried and subjected to natural sunlight for photocatalytic evaluation (Figure 4e). Repeated surface scan measurements of *x̅* RGB color histogram changes of a fixed area (5 cm^2^; after every 1 h until 6 h) showed the photocatalytic degradation of MV on the MPs via discoloration and the tendency to achieve the original parent color (i.e., dull white), which was clearly different for various MPs, as shown in Figures 4f,4g and S11. In T3- and T5-MPs, the rate of MV removal (discoloration) was high (79% ± 2) during 3 h, steadily increased between 3 and 5 h (84% ± 2), and slowed down between 5 and 6 h, with 85% ± 2 of total discoloration in 6 h. In the P25-MP, the discoloration was progressive with a maximum of 85% ± 2 achieved in 6 h. Interestingly, the T7-MP showed the best performance in MV photocatalytic removal, with higher discoloration during the first 3 h (93% ± 2) with steady and progressive discoloration between 3 and 6 h, reaching a maximum of 96% ± 2 in 6 h, a value close to complete discoloration (blank/dull white = 98% ± 2). In contrast, SOL-65, which adsorbed the maximum of MV (Figure 4d), failed to continue photocatalysis after 1 h (67% ± 2), which may be due to the larger particle size (≤150 μm) and titania agglomeration with siloxane resin reducing the active surface (see Figure S12). Possibly, TiO_2_-NPs degrade siloxanes rather than MV upon sunlight exposure.^99^ The natural sunlight photocatalytic activity of all MPs remained unchanged for a repeated cycle, which can be seen in Supporting Figure S13. In T3-, T5-, and P25-MPs, the rate of discoloration was progressive, reaching ≈82% ± 2 after 6 h. Once again, T7-MP was best with more robust discoloration in the first 3 h (89% ± 2) and steady and progressive discoloration between 3 and 6 h (93% ± 2). In a repeated cycle and due to lack of photocatalysis, MV tends to accumulate on SOL-65, showing 29% more intense values in the *x̅* RGB color histogram. Overall, T7-MP performed 10% ± 2 better for discoloration (removal of MV to reach the *x̅* of RGB color histogram value of parent dull white) than the other MPs. Moreover, minor differences over time in the control sample under sunlight may possibly affect MV light absorption in the visible part (λ 400–600 nm).

To differentiate the MP discoloration effect of natural sunlight and UV light, UV-assisted discoloration was also tested on all specimens at room temperature. The UV light accelerated the discoloration, evident from a detailed comparison in Supporting Figures S14 and S15. In particular, there was an ≈4% increase in the discoloration performance for T5- and P25-MP, while the discoloration rate for T7- and T3-MPs remained almost the same. For T7-MPs, further improvement was immeasurable, as almost all MV was already photodegraded under natural sunlight, achieving ≈96% discoloration.

**Figure 5:**
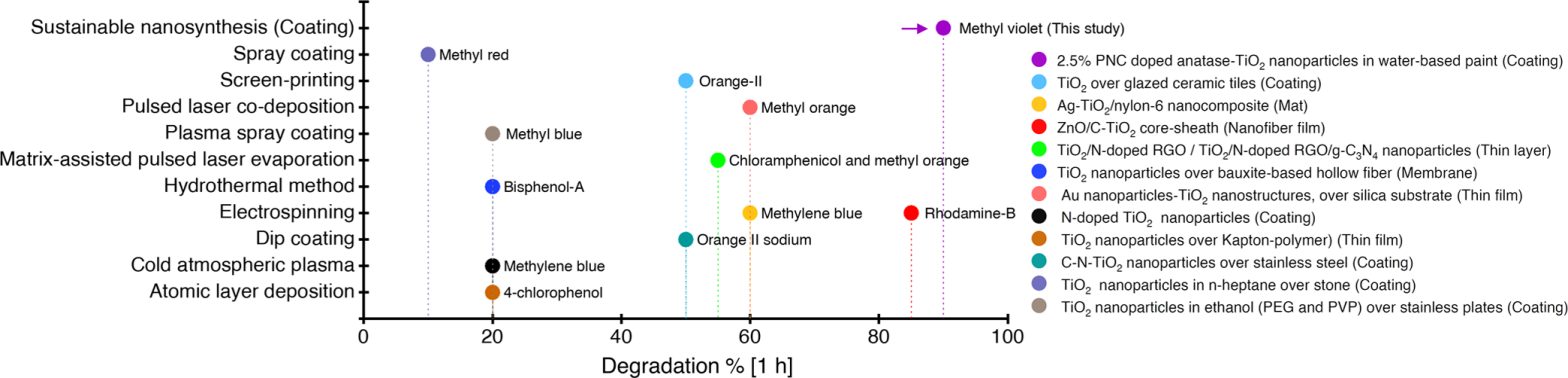
Photocatalytic performance of PNC-doped anatase TiO_2_-NPs as a photocatalytic additive to paint, compared to previously reported TiO_2_-based surface coating materials (see more details in Supporting Table S5).

A comparative analysis of photocatalytic activity of the T7-MP in terms of discoloration with materials reported in the literature is displayed in Figure 5 and Supporting Table S5. To enhance the photoactivity, TiO_2_-NPs were often used in the pure form^100^ in relatively high concentration (>50% in the paint polymer matrix)^101^ or modified with metals (e.g., Ag, ZnO, Au) or nonmetals (e.g., N, C, F, S) via different methods and then used as surface coatings of different substrates.^100,102−106^ Although the reported methods demonstrated better photocatalysis by modified TiO_2_-NPs, they came with limitations such as (i) nonsustainable consumption of natural resources to obtain raw materials for synthesis, (ii) complex synthesis procedures, (iii) costly setup restricting large-scale applicability, and most importantly (iv) photodegradation of the paint polymer due to active TiO_2_-NPs. The latter remains a challenging task, in particular, when modifying polymeric paints with TiO_2_-NPs.^4^

### Effect of TiO_2_-NPs on Paint Stability Analyzed by Micro-FTIR Spectroscopy

**Figure 6:**
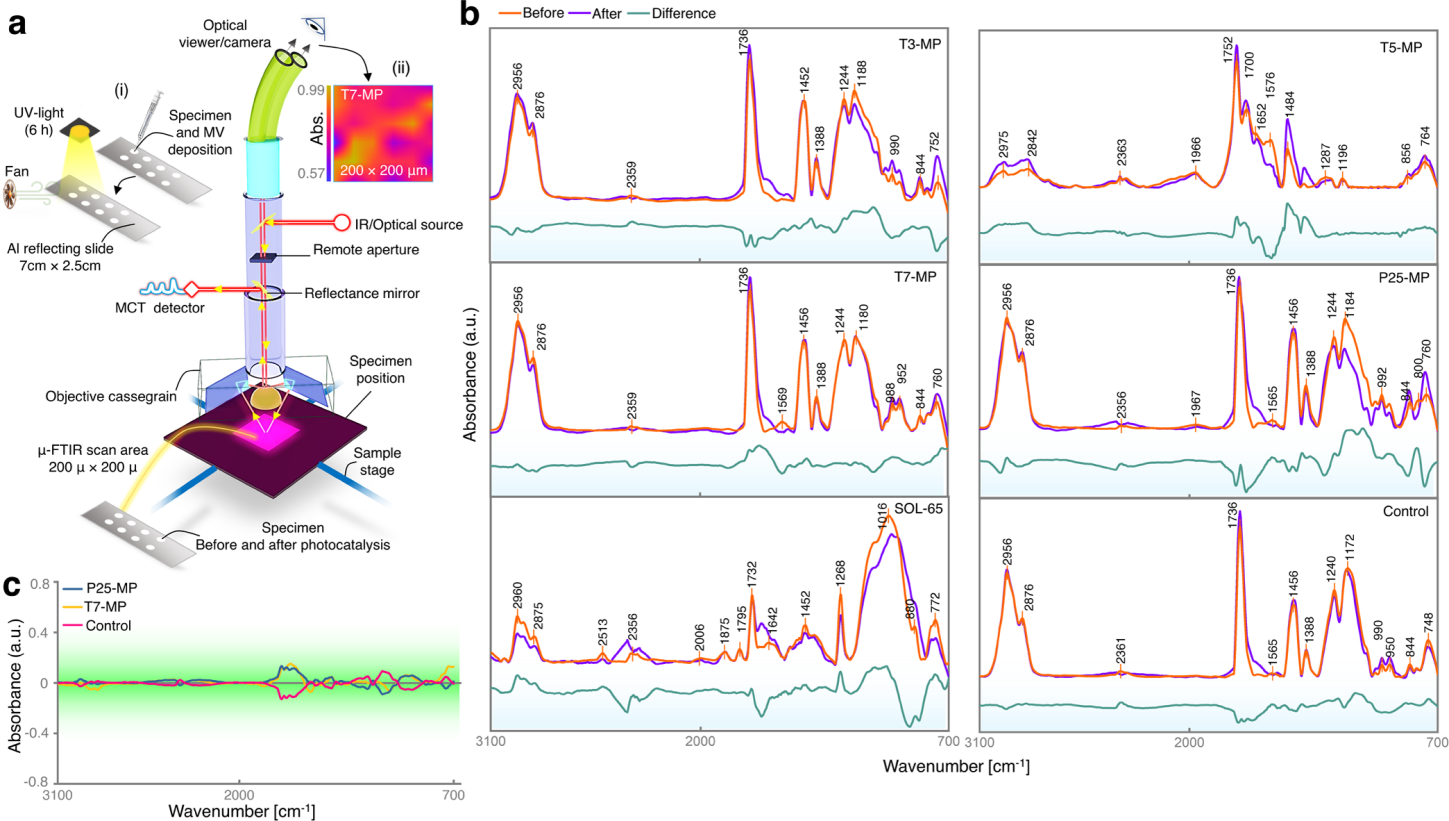
Stability of the paint polymer upon addition of PNC-doped TiO_2_-NPs. (a) (i) Experimental design showing modified paints deposited over an IR-grade Al slide (7 cm × 2.5 cm), drying at room temperature (in the dark for 24 h), exposure to MV (50 μL of 10 mg/L) for surface adsorption (drying in the dark for 6 h), and (ii) subjected to micro-FTIR analysis (homogeneous 200 μm × 200 μm area) before and after UV light (int. 15 ±1 W/m^2^), inducing photocatalysis. (b) Micro-FTIR, average spectrum (700–3100 cm^–1^) acquired from 200 μm × 200 μm over the sample with difference plots for MPs (T3-, T5-, T7-MP, P25-MP, and SOL-65) before and after photocatalysis. The control is the unmodified paint (for the correlation map and energy map, see Figures S16 and S17). (c) Difference plot (after minus before UV light-induced photocatalysis) for T7-MP, P25-MP, and control.

As mentioned, addition of TiO_2_-NPs to the polymeric paint matrix provides photoinduced self-cleaning properties, but this may also result in polymer degradation and release of hazardous volatile organic compounds (VOCs) due to photooxidation^107−109^ or TiO_2_-NP-mediated photocatalysis.^4,5,110^ In light of the promising photocatalytic performance by PNC-doped TiO_2_-NPs as an additive to water-based paints, it is crucial to investigate paint/TiO_2_-NP interactions, which was tackled via micro-FTIR analysis. MPs were deposited over an IR-grade aluminum slide, dried at room temperature, exposed to MV surface adsorption, and subjected to micro-FTIR analysis before and after UV light-induced photocatalysis, as shown in Figure 6a (see the Methods, Supporting Information).

The average spectra of MPs before and after photocatalytic tests are reported in Figure 6b. The related topographic distribution (correlation map, energy map with selected area micrograph) in which the colorimetric scale indicates the areas of higher (orange) or lower (blue) concentration can be seen in Figures S16 and S17. The correlation maps clearly highlight the homogeneity of all analyzed MPs.

For T3-MP, there were several modifications upon photocatalysis. As a result of photocatalytic degradation, water-based acrylic paints produced low-molecular weight molecules through Norrish type I or II reactions together by chain scissions in the early steps of the degradation process,^107^ which is obvious from a slight decrease in adsorption from 1000 to 1300 cm^–1^. In this region, 1188 cm^–1^ is assigned to C–O stretching, while 1244 cm^–1^ is attributed to asymmetric stretching vibrations of C–O–C bonds. The more significant increase in adsorption between 700 and 900 cm^–1^ is due to evolution of Ti–O bond vibrations, as a result of paint polymer (organic content) photodecomposition. This might have also contributed to the peak shift between 700 and 1500 cm^–1^. The sharp peak at 1736 cm^–1^ represents the carbonyl group of possibly methyl acrylate of the paint polymer, which becomes slightly broadened after photocatalysis, suggesting carbonyl oxidation. Moreover, as a result of photo-oxidation, H_2_O tends to accumulate over T3-MP, giving rise to νH–O–H (stretching vibrations) at 3500 cm^–1^. The peak between 800 and 900 cm^–1^, which is consistent in all the specimens, corresponds to aromatic C–H bending of VOCs, used as an additive to paint formulations.^111^ The peaks at 2876 and 2956 cm^–1^, corresponding to ν_s_(CH_3_) and ν_as_(CH_3_) bond vibrations, respectively, remained unaffected.

The T5-MP, T7-MP, and P25-MP exhibited properties quite similar to T3-MP. However, the difference shown in the 1000–1300 cm^–1^ region indicates more decomposition in the P25-MP than, e.g., in the T7-MP. The peak shift in the T7-MP and P25-MP between 700 and 1500 cm^–1^ is higher than for the T3-MP, suggesting different interactions of TiO_2_-NPs with the paint polymer matrix. Moreover, in SOL-65, the peaks representing the paint polymer, such as 1732 cm^–1^ (C=O of carbonyl group), 2875 cm^–1^ δ_s_(CH_3_), and 2960 cm^–1^ δ_as_(CH_3_), were less intense because of higher TiO_2_-NP concentration (≈15%) with bigger sized particles (>500 nm) (see Figure S12). The smaller amount of polymer over TiO_2_-NPs resulted in a greater degree of polymer decomposition upon photocatalysis. The broadness of peaks during photocatalysis also indicates that the TiO_2_-NPs induced degradation of MV and the polymer matrix, which might have resulted in accumulation of byproducts (such as carbonates, bicarbonates, etc.) over SOL-65.^112,113^ Overall, the T7-MP was the most stable modified composition (Figure 6c). The difference plot illustrates that the degree of paint polymer decomposition is in the order SOL-65 > P25-MP > T5-MP > T3-MP > T7-MP > control.

Finally, for ease of comparison, the physicochemical characterization data of all photocatalysts obtained via different methods are summarized in Supporting Table S6.

## Conclusions

Metal and organic wastes were used as potential raw materials to produce PNC-doped TiO_2_ nanoparticles (NPs). The use of sulfuric acid and high-temperature annealing may seem somewhat incompatible with the concept of green chemistry. However, when considering the broader context of heterogeneous waste reduction, resource conservation, and energy efficiency, the synthesis method presented herein follows most green chemistry principles, agreeing with the UN SDGs and US iWARM of the EPA. Doping by nitrogen phosphorus and carbon-related species during synthesis was confirmed by ATR-FTIR, EELS, EFTEM imaging, XPS, Raman spectroscopy, and DRIFTS. An optimum carbon content, along with P and N species, acted as strong inhibitors of e^–^/h^+^ recombination, decreasing it 7.3 times (T7-NPs) when compared to a standard P25 photocatalyst, as evident from 3D and synchronous photoluminescence (3D-SPL) spectroscopy analysis. The nonmetal dopants also hindered the anatase/rutile phase transformation, even after 700 °C calcination. When added to water-based polymeric paints, PNC-doped TiO_2_-NPs were able to photocatalytically remove 96% of the surface-adsorbed pollutants under natural sunlight and/or UV, paralleled by excellent stability in paint formulations, as confirmed by micro-FTIR surface analysis. The current results may serve as a basis for further field testing and commercial applications.

## Methods

### Synthesis Procedures

Green and sustainable nanosynthesis (SNS) through heterogeneous waste valorization of PNC-doped titanium oxide nanoparticles (TiO_2_-NPs) was achieved by the following steps, presented in Figure 1a.

The key modifications of a previously reported method^30^ are (i) alternative fallen leaf source as organic waste, (ii) water-based extract of fallen leaf powder, (ii) use of NaOH as a reducing agent, and (iv) calcination of oven-dried nanopellets up to 700 °C.

Briefly, fallen plant leaves were subjected to thorough washing with H_2_O and then ddH_2_O. To avoid the photodissociation of secondary metabolites, the washed leaves were shade-dried at room temperature (RT). Dried leaves were ground to make a fine powder. Next, 70 g of the ground leaf powder was soaked overnight in 1000 mL of ddH_2_O in a flask and then put on a hot-plate magnetic stirrer for 1 h at 70 °C and 300 rpm. Afterward, the whole suspension was filtered using Whatman filter paper No.1.

In a second step, the titanium metal scrap as metal waste was added to conc. H_2_SO_4_ (96%) in a round-bottom flask with a ratio of 2 g/100 mL at RT, to achieve metal leaching/dissolution.^114^ With this ratio, the H_2_SO_4_ was completely utilized so that the green synthesis process remains intact. After 24 h, the leaching/dissolution of titanium was further enhanced using ultrasonication. Subsequently, 100 mL of the dissolved Ti_2_(SO_4_)_3_ was further diluted in 100 mL of ddH_2_O. After this, the precipitation was completed with the displacement of SO_4_ with PO_4_, by a double displacement reaction between 200 mL of Ti_2_(SO_4_)_3_ and 200 mL of 1 M Na_3_PO_4_ at 80 °C for 1 h under continuous stirring at 150 rpm. The chemistry of the overall reaction can be understood from the following eq 1–3^115^

#### Step I


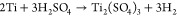
1
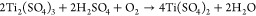
2

#### Step II


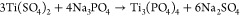
3500 mL of the prepared fallen leaf extract in a reaction flask was put on a hot-plate magnetic stirrer at 90 °C and 300 rpm. Subsequently, 21.4 g of Ti_3_(PO_4_)_4_·*x*H_2_O was added to the preheated extract and the reaction was continued for 2 h. During the reaction, the pH of the reaction mixture was adjusted to ∼5 by dropwise addition of 1 M NaOH solution. Thereafter, heating was discontinued, and the reaction mixture was allowed to cool to room temperature while stirring at 300 rpm. Later, PNC-doped TiO_2_-NPs were first collected by centrifugation at 8000 rpm for 20 min and then washed three times with ddH_2_O to get rid of uncoordinated secondary metabolites. Centrifuged PNC-doped TiO_2_-NPs were oven-dried at 90 °C overnight and then calcined at three different temperatures, which are 300, 500, and 700 °C, for 3 h, respectively.

Next, 2.5% (dry weight ratio) of as-prepared TiO_2_-NPs (T3-, T5-, T7-NPs) or P25 (commercial TiO_2_-NPs) was homogeneously mixed in water-based paints, respectively, called modified paints (MPs). The paint mix was applied over an inert substrate (wooden sheet) of 5 cm × 5 cm, precoated with a first layer of unmodified water-based paint. The thickness of the applied paint was confirmed through an Olympus BX51 microscope and was found to be ≈70 μm and homogeneous (Figure 1a, right). Before further use, the prepared specimens were first dried at RT for 24 h and then dried in a desiccator in the dark for another 24 h (for more details, see the Supporting Information).

### Characterization of Materials

The mechanism of the heterogeneous waste (industrial metal waste and fallen leaf extract)-derived synthesis of PNC-doped TiO_2_-NPs was investigated by ATR-FTIR.

X-ray diffraction (XRD) was carried out at RT using a Cu Kα radiation source (λ = 1.5406 Å) at an operating voltage of 40 kV (current of 30 mA). Crystallographic parameters of the prepared materials were identified through Rietveld refinement using GSAS-II (v4776) and OriginPro (v2021).

The morphology and crystal structure were evaluated by high-resolution transmission electron microscopy (HR-TEM), electron energy loss spectrometry (EELS), and selected area electron diffraction (SAED), using a FEI TECNAI F20 field emission microscope equipped with a GATAN GIF Tridiem energy filter and a GATAN Rio16 CMOS camera.

Surface chemistry of PNC-doped TiO_2_-NPs was studied using a DRIFTS cell.^116^ The IR spectrometer was equipped with a silicon carbide IR source (Globar), a liquid nitrogen-cooled mercury cadmium telluride (MCT) detector, and a commercial DRIFTS mirror unit.

X-ray photoelectron spectroscopy (XPS) measurements were performed with a Specs XR50© high-intensity nonmonochromatic Al/Mg dual anode and an X-ray source Phoibos 100 energy analyzer (EA) with a multichannel plate.

PNC doping of TiO_2_-NPs was further confirmed using confocal micro-Raman spectroscopy at room temperature. The Raman system was equipped with a Nikon Eclipse TiU optical microscope and thermoelectrically cooled charge-coupled device (CCD) detector. The excitation source used was a diode laser (532 nm). Raman spectra were recorded between 0 and 3000 cm^–1^.

Thermogravimetry (TG), derivative thermogravimetry (DTG), and simultaneous differential thermal analysis (SDTA) of PNC-doped TiO_2_ were used to monitor the thermal properties in terms of mass change and exothermicity/endothermicity, both in air and nitrogen.

The band gap 3D photoluminescence (3D -PL) and synchronous PL (SPL) of T3-, T5-, T7-NPs, and P25 (100 ppm in ddH_2_O) were studied at room temperature by a Shimadzu RF-600 spectrofluorophotometer (validated LOD= 1 × 10^–13^ mol/L), at a scan rate of 20,000 nm/min. 3D PL spectroscopy (excitation–emission spectrum contour plots) was measured with luminescence intensity as a function of excitation wavelength (λ_Ex_ = 280–450 nm) and emission wavelength (λ_Em_= 500–900 nm). For the SPL scan mode, samples were simultaneously scanned using both an excitation monochromator and a fluorescence monochromator that are offset by fixed wavelength intervals of 200 nm (Δλ = λ_Em_ – λ_Ex_ = 200 nm), λ_Ex_ starting from 300 nm and λ_Em_ from 500–900 nm, and at measurement intervals of 0, 50, and 100 s. At the end, the average of SPL plots was used for interpretation (for more details, see the Supporting Information).

### Performance Evaluation of the TiO_2_-NP Additive Photocatalytic Paint

The photocatalytic performance of the MPs is expressed as the rate of removal of adsorbed pollutants over the paint surface and tendency of the paint surface to achieve the original primary color (which is dull white). A customized setup was constructed that allowed continuous monitoring of pollutant (for example, methyl violet—MV) surface adsorption via the change in concentration vs time. The customized setup is well elaborated in Figure 4b and the Supporting Information.

The amount of dye adsorbed on the surface of MPs, measured through the setup described above, also corresponds to the color intensity or average (*x̅*) RGB color histogram of a sample, which is the combined *x̅* value of RGB (red, green, and blue) color standards (RGB standard defines each color as a combination of red, green, and blue values). Thus, the *x̅* RGB color histogram can be defined as
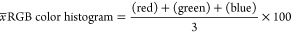
4

Since the parent color of the MP surface is dull white, it corresponds to the *x̅* RGB color histogram of the maximum value, which is 250 ± 3. On the other hand, any deviation from the dull white color will change the value in the reverse order (magnitude of 250 ± 3 for dull white and 0 for black). The higher the dye adsorption, the more intense the color will be and the lower the *x̅* RGB color histogram value is and vice versa (e.g., Figure S11). Accordingly, the photocatalysis rate of the specimen is directly proportional to the rate of discoloration.

The adsorbed pollutant (dye) removal capacity of all MPs was measured under natural sunlight and UV light. The irradiance intensity of natural sunlight on the sunny days was measured for the visible range = 465–480 W/m^2^ (using a Delta Ohm HD 2101.1 photo-radiometer equipped with visible sensor LP 471 RAD 400 nm–1050 nm) and the UV range = 6.1–6.4 W/m^2^ (by UVA sensor LP 471 UVA RAD 315–400 nm). UV light experiments were conducted at room temperature using a UV halogen lamp (400W), with a measured light intensity of 15 ± 1 W/m^2^ (UVA sensor LP 471 UVA RAD 315–400 nm). The color changes over the MPs were measured for each 1 h until 6 h, through processing of high-resolution (pixels= 600 dpi) surface scanner (HP officeJet 4500) data of the same area (pixel area of 5 cm^2^, complete contact with the scanner plate to avoid illumination inconsistency errors for RGB measurements) by ImageJ (for more details, see the Supporting Information).

### TiO_2_-NP–Paint Interactions and Degree of Polymer Degradation Analysis

To investigate TiO_2_-NPs versus paint interactions and the stability of the whole complex before and after photocatalysis of adsorbed MV, 50 μL of as-prepared MPs was first spread over a 7 cm × 2.5 cm aluminum (Al) slide and then dried in the dark for 24 h. Next, 50 μL of 10 mg/L MV solution was poured over each specimen, dried in the dark for 6 h, and subjected to room-temperature UV light (int. 15 ±1 W/m^2^)-induced photocatalysis (in terms of discoloration) for 6 h. Micro-FTIR data were recorded for all specimens before and after UV light-induced photocatalysis, as shown in Figure 6a. Infrared microscopy spectra were collected by a Spectrum GX 1 FT-IR spectrometer equipped with an Autoimage microscope (with a photoconductive HgCdTe, MCT, array detector, operating at liquid N_2_ temperature), in the range from 4000 to 700 cm^–1^ (for more details, see the Supporting Information).

## Data Availability

The data that support the findings of this study are available from the corresponding author upon reasonable request. Correspondence and requests for materials should be addressed to G.R. Source data are provided with this paper.

## Supporting Information Available

Material description, experimental procedures, XPS C 1s region analysis, thermal analysis, XRD, HRTEM, SAED, XPS, Raman spectroscopy, TG/DTG/SDTA, photoluminescence, band gap energy, SEM with EDXS, UV adsorption, discoloration analysis, topographic distribution, energy and correlation map, crystal parameters and elemental composition of NPs, SAED with phase identification, thermal properties, comparison of photocatalytic performance, and summary of physicochemical characterization (PDF)
