# Supplementary material for: Highly Stable Self-Cleaning Paints Based on Waste-Valorized PNC-Doped TiO2 Nanoparticles
Source: ACS Catal. 2024 Mar 15;14(7):4820–34. doi: 10.1021/acscatal.3c06203 (PMC11003396; doi:10.1021/acscatal.3c06203)
Supplement: Supplementary file 1 — cs3c06203_si_001.pdf [file cs3c06203_si_001.pdf]

Supporting Information for

Highly stable self-cleaning paints based on waste-valorized PNC-doped TiO_2 nanoparticles

Qaisar Maqbool[1,2], Orlando Favoni[1], Thomas Wicht[2], Niusha Lasemi[2], Simona Sabbatini[1], Michael Stöger-Pollach[3], Maria Letizia Ruello[1], Francesca Tittarelli[1], Günther Rupprechter[2]*

[1]Department of Materials, Environmental Sciences and Urban Planning (SIMAU), Università Politecnica delle Marche, INSTM Research Unit, via Brecce Bianche 12, 60131 Ancona, Italy.

[2]Institute of Materials Chemistry, TU Wien, Getreidemarkt 9/BC, A-1060 Vienna, Austria.

[3]University Service Center for Transmission Electron Microscopy, TU Wien, Wiedner Hauptstr. 8-10, 1040 Vienna, Austria.

* Correspondence to guenther.rupprechter@tuwien.ac.at

This PDF file includes:

Materials and Methods
Supporting Text S1 and S2
Figs. S1 to S17
Tables S1 to S6

Materials

Titanium metal scrap (thankfully provided by CALEFFI™ s.r.l Italy), inert substrate (wooden sheet, dimension: 5 cm × 5 cm), H_2SO_4 (96 %), commercial grade multi-surface water based paint (white color-50, BORMA WACHS™ s.r.l), Na_3PO_4, (Sigma Aldrich™), sterile ultra-pure water (ddH_2O) by Milli-Q™, fallen leaves as a potential organic waste (Parco Della Cittadella di Ancona, Italy), Methyl violet 2B (CAS No: 8004-87-3, by Sigma-Aldrich™), scanning electron microscope (SEM) coupled with energy-dispersive X-ray spectroscopy (EDXS) (ZEISS SUPRA™-40 and X-Flash Detector 410-M by Bruker™ Nano GmbH), high resolution transmission electron microscopy (HR-TEM) by a FEI TECNAI F20, Olympus™ BX51 microscope, confocal micro-Raman spectroscope (Horiba XploRA™), micro-FTIR Perkin–Elmer™ Spectrum GX 1 FT-IR spectrometer equipped with a Perkin–Elmer™ autoimage microscope, SDTA– TGA (Mettler™-851), centrifuge (HERMLE™ Z380), diffuse reflectance infrared Fourier transform spectroscopy (DRIFTS) using a Bruker Vertex 70 spectrometer with a DRIFTS cell (Pike™), solar lamp (OSRAM™-Ultra-vitalux-300W- 230AC), hotplate magnetic stirrer (RH basic, IKA™, Germany), water bath ultrasonication (FALC™, Italy), heating oven (LA PORTA™, Italy), Electric Grinder (KENWOOD™ 500W), rotary vacuum evaporator (Model=R-10, BUCHI™, Switzerland), centrifuge (EC-CL31R Multispeed, Thermo Scientific™, USA), water bath ultrasonication (FALC™, Italy), heating oven (LAPORTA™, Italy), X-ray powder diffractometer (Bruker™), TGA (Mettler™-851), UV/Vis spectrometer (Shimadzu™ UV-1900i), Florescence spectrometer (Shimadzu™ RF-6000i).

Methods

Synthesis procedures

Green and sustainable nanosynthesis (SNS) of phosphate, nitrogen and carbon (PNC) doped titanium oxide nanoparticles (TiO_2-NPs) through heterogenous waste valorization was achieved following a previously reported, but significantly modified, method [1]. The major modifications include (a) alternative fallen leaves source as potential organic waste, (b) water-based extract of fallen leaves powder, (c) use of NaOH as reducing agent and (d) calcination of oven dried nanopellets up to 700 °C.

Briefly, the SNS of TiO_2-NPs, and modification of multisurface water-based paint was accomplished by the following five steps, presented in Fig. 1a.

i. The processing of fallen plant leaves as potential organic waste and recovery of the final extract was accomplished following our previously reported methods with modifications [2]. Fallen leaves were harvested in November. To ensure the repeatability among different synthesis batches, it is important to highlight here that fallen leaves should be collected only at a specific time of the year to match the metabolic profile and stored at room temperature.

Briefly, to get rid of undesired soil particles, fallen plants were subjected to thorough washing with H_2O, then ddH_2O. To avoid the photodissociation of secondary metabolites, the washed leaves were shade dried at room temperature (RT). Dried leaves were ground to make a fine powder. Next, 70 g of the grinded leaves powder was soaked overnight in 1000 mL of ddH_2O in a flask and then put on a hot plate magnetic stirrer for 1 h at 70 °C and 300 rpm. Thereafter, the whole suspension was filtered using Whatman filter paper No.1. The residue (biomass) recovered over the filter paper was consider as a potential by-product for nanocellulose extraction [1] for ongoing research.

ii. In the second step, the titanium metal scrap as a potential metal waste was added to conc. H_2SO_4 (96%) in a round bottom flask with the ratio of 2 g / 100 mL at RT, to achieve metal leaching/dissolution [3]. With this ratio, the H_2SO_4 was completely utilized so that the green synthesis process remained intact. After 24 h, the leaching/dissolution of titanium was further enhanced using ultrasonication. Subsequent, 100 mL of the dissolved $Ti_2(SO_4)_3$ was further diluted in 100 mL of ddH_2O. After this, the precipitation was completed with the displacement of SO_4 by PO_4, by double displacement reaction between 200 mL of $Ti_2(SO_4)_3$ with 200 mL of 1M Na_3PO_4 at 80 °C for 1 h under continuous stirring at 150 rpm. The chemistry of the overall reaction can be understood from the following equations [4],

Step-I

$$2\ Ti + 3\ H_2SO_4 \rightarrow Ti_2(SO_4)_3 + 3\ H_2 \quad \text{(S1)}$$

$$2\ Ti_2(SO_4)_3 + 2\ H_2SO_4 + O_2 \rightarrow 4\ Ti(SO_4)_2 + 2\ H_2O \quad \text{(S2)}$$

Step-II

$$3\ Ti(SO_4)_2 + 4\ Na_3PO_4 \rightarrow Ti_3(PO_4)_4 + 6\ Na_2SO_4 \quad \text{(S3)}$$

iii. 500 mL of the prepared fallen leaves extract in a reaction flask was put on a hot plate magnetic stirrer at 90 °C and 300 rpm. Subsequently, 21.4 g of $Ti_3(PO_4)_4 . xH_2O$ was added to the preheated extract and the reaction was continued for 2 h. During the reaction, the pH of the reaction mixture was adjusted to ~5 by drop-wise addition of 1M NaOH solution. Thereafter, heating was discontinued, and the reaction mixture was allowed to cool to room temperature while stirring at 300 rpm. Later, PNC-doped TiO_2-NPs were first collected by centrifugation at 8000 rpm for 20 min then washed three times with ddH_2O to get rid of uncoordinated secondary metabolites. The centrifuged PNC-doped TiO_2-NPs were oven dried at 90 °C overnight and then calcined at three different temperatures, which is 300, 500 and 700 °C for 3 h, respectively.

The labels after calcination are,

- T3-NPs (300 °C),
- T5-NPs (500 °C) and

– T7-NPs (700 °C).

iv. Next, 2.5% (dry weight ratio) of as prepared TiO_2-NPs (T3-, T5-, T7-NPs) or P25 (commercial TiO_2-NPs) were homogenously mixed in water-based paint, respectively. For mixing, NPs and paint were added in a 50 mL glass vial, closed with a lid and stirred (using 1 cm stirrer) over a magnetic stirrer for 15 minutes.

Labels of the modified samples are,

– T3-MP (T3-NPs as an additive to modify water-based paint by BORMA WACHS® s.r.l)

– T5-MP (T5-NPs as an additive to modify water-based paint by BORMA WACHS® s.r.l)

– T7-MP (T7-NPs as an additive to modify water-based paint by BORMA WACHS® s.r.l)

– P25-MP (commercial P25 TiO_2-NPs as an additive to modify water-based paint by BORMA WACHS® s.r.l)

– SOL-65 (SOLARYA 65, a commercial photocatalytic paint by BOERO® s.r.l Italy)

– Control (unmodified water-based paint by BORMA WACHS® s.r.l with adsorption/photocatalytic test, a positive control)

– Blank (unmodified water-based paint by BORMA WACHS® s.r.l without adsorption or photocatalytic test). In our case "blank" is not a performing sample, but the sample without MV exposure, representative of the maximum value which can be achieved as a result of photocatalysis.

v. The paint mix after preparation (as stated in step iv) was applied using brushless flow coating over an inert substrate (wooden sheet) of 5×5 cm, precoated with a first layer of unmodified water-based paint. Brushless flow coating was employed to minimize the formation of brush marks or uneven areas. Briefly, for homogenous surface preparation by brushless flow coating, the paint mix was poured over a horizontally aligned wooden substrate (5×5 cm) surface and spread using a non-adhesive microscope glass slide. Wood was preferred over metal, because of good wood capacity to dissipate heat under sunlight or UV light and to avoid a direct heating effect during photocatalysis. The thickness of the applied paint was confirmed through an Olympus® BX51 microscope and was found to be ≈70 µm and homogenous (Fig. 1a, right). Before further use, the prepared specimens were first dried at RT for 24 h, then dried in a desiccator in dark for further 24 h.

Characterization of materials

Mechanistic study of green synthesis through "attenuated total reflectance-Fourier transform infrared (ATR-FTIR) spectroscopy" of the extract

The mechanism of the heterogenous waste (industrial metal waste and fallen leave extract) derived synthesis of PNC-doped TiO_2-NPs was investigated by Attenuated Total Reflectance-Fourier Transform Infrared (ATR-FTIR) spectroscopy in air with 32 scans and a spectral resolution of 4 cm^{-1}. Briefly, 1 µL of the water extract before the synthesis and after the synthesis procedure was taken and diluted in 9 µL of EtOH. One drop of the prepared solution was poured over a ZnSe crystal and IR absorption spectra were recorded in reflectance mode in the range 550 to 4000 cm^{-1}. During the IR measurements, (9:1, EtOH/H_2O) was taken as background. Baseline correction (polynomial line fit) and smoothing were performed in all cases.

Crystal structure and morphology

X-ray diffraction (XRD) was carried out at RT using a Cu-Kα radiation source (λ = 1.5406 Å) at an operating voltage of 40 kV (current of 30 mA). The XRD measurements were recorded at the angle of diffraction (2θ) between 10° and 85°. Crystallographic parameters of the prepared materials were identified through Rietveld refinement using GSAS®-II (v4776) and OriginPro® (v2021). The average crystallite size was measured using Debye-Scherrer's equation,

$$D = 0.9\lambda/\beta\cos\theta \quad \text{(S4)}$$

where D is the average crystallite size (nm), λ is X-ray wavelength (1.5406 Å), β is the full width at half maximum (FWHM) in radians, and θ is Bragg's angle.

The morphology and crystal structure of the different materials (T3-NPs, T5-NPs, T7-NPs, P25) were evaluated by high resolution-transmission electron microscopy (HR-TEM), electron energy loss spectrometry (EELS) and selected area electron diffraction (SAED), using a FEI TECNAI F20 field emission microscope equipped with a GATAN GIF Tridiem energy filter and a GATAN Rio16 CMOS camera. The Inorganic Material Database (AtomWork) [5] was used to obtain crystallographic data and phase identification of T7-NPs. For HR-TEM, samples were prepared as follows: 5 mg T3-, T5-, T7-NPs or P25 and 5 mL diH_2O were ultrasonicated for 10 min. Using a micropipette, 1 drop of the ultrasonicated suspension was transferred on a commercial TEM copper grid covered with a lacy carbon film. Before each measurement, the TEM sample was vacuum dried for 15 min.

Diffuse reflectance infrared Fourier transform spectroscopy (DRIFTS)

The synthesized materials (*e.g.* T3-, T5-, T7-NPs) were analyzed using a DRIFTS cell [6]. The IR spectrometer was equipped with a silicon carbide IR source (Globar®), a liquid nitrogen-cooled mercury cadmium telluride (MCT) detector and a commercial DRIFTS mirror unit. Briefly, 50 mg of NPs material was placed in a porous ceramic sample cup and mounted in the DRIFTS cell. Spectra of air serving as background were also acquired. IR-spectra (256 scans) were recorded *via* the OPUS® (v6.5) software. Baseline (polynomial line fit) and smoothing were performed in all cases.

X-ray photoelectron spectroscopy (XPS)

X-ray photoelectron spectroscopy (XPS) measurements were performed with a Specs XR50© high intensity non-monochromatic Al/Mg dual anode and an X-ray source Phoibos 100 energy analyzer (EA) with multichannel plate. All spectra were obtained at RT, at an emission angle of 0° and a pass energy of 20 eV, using an Al anode with Kα radiation at 1486.6 eV. Data analysis was performed via CasaXPS. As the nature of adventitious carbon species may change due to the different heat treatments, the samples were instead calibrated keeping the Ti 2p binding energy fixed at 459.3 eV[7] for TiO_2. All spectra were analyzed with an energy step width of 0.1 eV, but for clarity only every second or third data point is shown in the figures.

Raman spectroscopy

A Horiba XploRATM INV was used for confocal micro-Raman spectroscopy at room temperature. The Raman system was equipped with a Nikon Eclipse TiU optical microscope, motorized XY stage and thermoelectrically cooled charge-coupled device (CCD) detector. The excitation source used was a diode laser (532 nm). In order to avoid phase changes with laser irradiation, the laser was operated at 25% intensity. The laser beam was focused on the sample by means of a 40x dry objective.

Raman spectra were recorded between 0 and 3000 cm^{-1} using a holographic grating with 1800 grooves/mm, acquisition time of 1 s, accumulation of 30 measurements, slit of 200 μm and hole of 300 μm. The LapSpec6 spectroscopy suite software from HORIBA Scientific was used for calibration and collection of spectra. μ-dish holders (Ibidi) with a glass bottom and a diameter of 35 mm were used for the micro-Raman analysis of P25 and PNC-doped TiO_2 nanoparticles.

Thermal stability analysis

Thermogravimetry (TG), derivative thermogravimetry (DTG) and simultaneous differential thermal analysis (SDTA) of PNC doped TiO_2 (T3-, T5-, T7-NPs) and P25 were used to monitor the thermal properties in terms of mass change and exothermicity/endothermicity, both in air and nitrogen. The gas flow was set to 60 mL/min in the temperature range of 25–1000 °C, for P25, T3-NPs, T5-NPs, and until 1300°C for T7-NPs, with a rate of 10 °C/min. For each sample, 5- 20 mg of the dried powder sample were measured in an alumina-150 μL sample holder.

3D photoluminescence spectroscopy

The band gap 3D-photoluminescence (3D-PL) and synchronous PL (SPL) of T3-, T5-, T7-NPs and P25 (100ppm in ddH_2O) were studied at room temperature by a Shimadzu™ RF-600 spectrofluorophotometer (validated LOD= 1×10^{-13} mol/L), at scan rate of 20000 nm/min. 3D-PL spectroscopy (excitation-emission spectrum contour plots) was measured with luminescence intensity as a function of excitation wavelength (λ_{Ex}=280 to 450 nm) and emission wavelength (λ_{Em}= 500 to 900 nm). While for the SPL scan mode, samples were simultaneously scanned using both an excitation monochromator and a fluorescence monochromator that are offset by fixed wavelength intervals of 200 nm ($\Delta\lambda = \lambda_{Em} - \lambda_{Ex} = 200$ nm), λ_{Ex} starting from 300 nm and λ_{Em} from 500 – 900 nm and at measurement intervals of 0, 50 and 100 seconds. At the end, the average of SPL plots were used for interpretation. All the 3D-PL spectra were acquired in automated correction mode (software= LabSolutions RF™). The ddH_2O (background) was subtracted from all the 3D-PL spectra. Deconvolution of SPL spectra were performed in OriginPro® (plugin "Peak Deconvolution v1.90" with Gaussian fit function, until best R^2 were reached). Baseline correction (polynomial line fit) and smoothing were performed in all cases.

Performance evaluation of TiO_2-NPs additive photocatalytic paint

Pollutant (Methyl violet 2B) adsorption and quantification through online UV-Vis–spectroscopy

The photocatalytic performance of the MPs is expressed as rate of removal of adsorbed pollutants over the paint surface and tendency of the paint surface to achieve the primary colour (which is dull white). This requires simulating a highly aggressive environment over the photocatalytic paint surface, which can be achieved via pollutant adsorption (for example, Methyl violet 2B - MV). Accordingly, a customized setup was constructed that allowed continuous monitoring of pollutant surface adsorption via the change in concentration vs time. The customized setup is well elaborated in Fig. 4b. Briefly, it consisted of the following components,

a) A UV/Vis spectrometer (Shimadzu® UV-1900i) with a commercial test tube holder.

b) A test tube (⌀) 22 mm, ℓ =115 mm, connected from the top with two high performance, pressure, and chemical resistant silicon tubes (inner ⌀ 1.6 mm). Both tubes were inserted inside the test tube through a test tube lid. This adjustment was achieved by making two pores in the rubber lid and sealing after crossing silicone tubes. The extension of both silicone tubes inside the test tube were adjusted in such a way that after adjustment, the silicon tubes will not obscure the detection window of the spectrophotometer. The silicone tubes inside the test tube should be airtight upon assembly and were outside connected to a peristaltic pump (30 mL/min) at one end.

c) 100 ml of dye solution (10mg/L) were placed in a 180 mm round bottom borosilicate glass beaker. A tripod stand was immersed inside the dye solution. The top of the tripod stand, with

the horizontal level close to the glass beaker opening edges, was used to hold the MP with the paint surface facing upward. The other end of the silicone tubes, as described in (b), were adjusted in such a way that one tube submerged in the dye solution and other was suspended at the top of the MP (to expose the sample surface to a continuous dye flow).

d) The cyclic flow of the dye solution over the MP was regulated via the peristaltic pump (connected to silicon tube) in the direction from dye solution → spectrophotometer detector → MP surface → dye solution.

The components (a), (b), (c) and (d), when operated collectively, can then be used to perform online UV-Vis spectroscopic absorption measurements (see Fig. 4b for a sketch).

The flow rate of the dye solution over the MP surface was adjusted to 30 mL/min, which always allows formation of a 4 mL dye solution suspension over the MP surface. This 4 mL of the dye solution layer over the MP is continuously refreshed, hence allowing a nonstop cyclic flow of the dye solution over the MP surface. The whole setup was well calibrated, and it was realized that with a continued flow rate of 30 mL/min, any change in the concentration of dye solution will be fully read by the spectrophotometer detector in 3.33 min., as shown in Fig. 4b (right).

Using the above-described setup, the adsorption of pollutant (MV) over T3-MP, T5-MP, T7-MP, P25-MP, and SOL-65 was monitored at the flow rate of 30 mL/min over the surface, purged for 1 h, with an automated spectrophotometer scan rate of 60/h. Baseline correction, blank subtraction, and calibration (of at least 4 different concentrations of dye solution) were performed for all UV-Vis experiments (software "LabSolutions UV-Vis v1.12" by SHIMADZU®).

Correlation between dye adsorption versus average color histogram

The amount of dye adsorbed on the surface of MPs, measured through the setup described above, also corresponds to the color intensity or average (x̄) RGB color histogram of a sample, which is the combined x̄ value of RGB (red, green and blue) color standards (RGB standard define each color as a combination of red, green and blue values). So, the x̄ RGB color histogram can be defined as,

$$\bar{x}\ RGB\ color\ histogram = \frac{\text{(Red)} + \text{(Green)} + \text{(Blue)}}{3} \times 100 \quad \ldots\ldots\ldots \text{(S5)}$$

Since the original parent color of the MP surface is dull white, it corresponds to x̄ RGB color histogram of the maximum value, which is 250±3. On the other hand, any deviation from the dull white color will change the value in reverse order (magnitude of 250±3 for dull white and 0 for black). The higher the dye adsorption, the more intense will be the color and the lower will be the x̄ RGB color histogram values, and *vice versa* (e.g.,

Fig. S11). Considering this, the rate of photocatalysis over the specimen is directly proportional to the rate of discoloration.

Photocatalysis under natural sunlight and UV light

At first, the adsorbed pollutant (dye) removal capacity of all the MPs were measured under natural sunlight. The irradiance intensity of natural sunlight on the sunny days at the Monte Dago Campus of the Università Politecnica delle Marche, Ancona, Italy was measured for the visible range = 465-480 W/m^2 (using a Delta Ohm HD 2101.1 photo radiometer equipped with visible sensor LP 471 RAD 400nm-1050nm) and the UV range =6.1-6.4 W/m^2 (by UVA sensor LP 471 UVA RAD 315-400 nm). The recorded values were close to the standardized solar irradiation in the period of August-September at the experimental site [8]. The exposure time was selected with starting daytime from 10:00 h, when solar elevation reached 45° till 16:00 h, as shown in Fig. 4e. UV-light experiments were conducted at room temperature using a UV halogen lamp (400W), measured light intensity of 15±1 W/m^2 (UVA sensor LP 471 UVA RAD 315-400 nm). The color changes over the MPs were measured each 1 h till 6 h, through processing of high-resolution (pixels= 600 dpi) surface scanner (HP™ officeJet 4500) data of the same area (pixel area of ≈5 cm^2, complete contact with scanner plate to minimize illumination inconsistency errors for RGB measurements) by imageJ™.

TiO_2-NPs-paint interactions, and degree of polymer degradation analysis by micro- Fourier Transform Infrared (Micro-FTIR) spectroscopy

To investigate TiO_2-NPs versus paint interactions and stability of the whole complex before and after photocatalysis of absorbed MV, 50 µL of as prepared MPs (T3-MP, T5-MP, T7-MP, P25-MP and SOL-65) were first spread over a 7 cm × 2.5 cm aluminium (Al) slide, then dried in dark for 24 h. Next, 50 µL of 10 mg /L of MV solution was poured over each specimen, dried in dark for 6 h, and subjected to room temperature UV-light (int. 15±1 W/m^2) inducing photocatalysis (in terms of discoloration) for 6 h. Micro-FTIR data were recorded for all deposited specimens before and after UV-light induced photocatalysis, as shown in Fig. 6a. Infrared Microscopy spectra were collected by a Spectrum GX 1 FT-IR spectrometer equipped with an autoimage microscope (with a photoconductive HgCdTe, MCT, array detector, operating at liquid N_2 temperature), in the range from 4000 to 700 cm^{-1}. Absorption spectra, as result of 32 scans, were collected on all specimens deposited on IR grade Al slide. The spectral and spatial resolution were 4 cm^{-1} and 200 x 200 μm^2, respectively. Background spectra were acquired on clean regions of the Al mirror. Specific areas of interest were identified by means of a microscope television camera. Baseline correction (polynomial line fit) and smoothing were performed in all cases. All spectra were scaled for equal intensity in the C-O band absorption (1059 cm^{-1}). Assignment of bands was following literature data [9].

Supporting Text

S1: XPS C1s region analysis

Interestingly, P25 showed a surprisingly high carbon content of ~20.6 %, with the main peak at ~285 eV and two additional species CI at ~287 eV and CII at ~289 eV (see Fig. S3 and Table S3). Therefore, the pure copper tape used to mount the samples was measured to evaluate its possible contribution to the C1s intensity. As the copper tape (3M Copper Conducting Tape 1182) is covered by a layer of acrylic adhesive, its contribution can only be measured via the C1s (and in smaller amounts via the O1s) region. A comparison of the C1s spectra of P25 and the copper tape shows great similarities, suggesting that a significant portion of the C1s intensity of the P25 sample may actually originate from the copper tape. Subtracting this contribution (via constraining the peaks according to the fit of the pure copper tape) would lower the carbon content of P25 to roughly 7 % of adventitious carbon. Considering the small peaks at ~289 eV present both for T3 and T5 it is possible that there's also some contribution of the copper tape to these spectra. The peak may also originate from oxidized carbon species (e.g., C=O) of the organic materials (compare DRIFTS). Furthermore, for all modified NPs the main C1s peak was shifted to lower BE values of ~284.4 eV, typical for graphitic sp^2 carbon, [10] as expected for the freshly calcined samples. We thus expect a low contribution of the copper tape to the C1s spectra of the doped NPs. That said, due to the given uncertainties, we refer to the results of the complementary characterization methods (DRIFTS, Raman, TGA) concerning both the qualitative and quantitative analysis of carbon species.

S1: Thermal analysis (TG/DTG/SDTA)

Commercial P25 as reference and the prepared NMs T3-, T5-, and T7-NPs were analyzed through thermal TG/DTG/SDTA analysis, as shown in Fig. S6 and supporting table S4, to assess the crystalline stability. For each specimen, the main temperature intervals (ΔTn) with the corresponding weight loss and temperatures of maximum decomposition (Tn), if observed, are reported in supporting table S4.

Generally, under N_2 the thermal behavior of TiO_2-NPs can roughly be divided into three stages. The first endothermic stage from room temperature to about 200 °C can be attributed to the evaporation of small molecules such as physically adsorbed water (dehydration) and removal of organic solvent residues. The further weight loss from approximately 200 to 600°C, with a global exothermic behavior, may include the TiO_2 phase transformation from amorphous to anatase [11], thermal decomposition of the peroxo titanium [12,13] and the decomposition of carbon-based compounds. Beyond approximately 600 °C, the endothermic peak in the DTA curve without no weight loss reflects the TiO_2 phase transformation from anatase to rutile [11,14,15].

In particular, for commercial P25, under N_2, the first endothermic 0.6% weight loss from 35 to 125 °C (ΔT1), ascribed to the loss of surface adsorbed water molecules, has maximum weight losses at T = 40 and 120 °C. Further increase in temperature results in globally exothermal 0.8% weight loss until T = 567° (ΔT2 + ΔT3). Moreover, at a very high temperature (T>600 ◦C) and reducing environment, there is a possible pyrolytic

carbon incorporation into the TiO_x crystal matrix, augmenting sample weight (+0.2%). In total, P25 shows 1.2% weight loss under N_2. The same sample in air shows similar thermal behavior, but with less evident endothermic peaks, since the presence of oxygen favors the exothermic processes. At the start, 0.7% weight loss due to water was observed with maximum at 40 °C (ΔT1). Then, the global endothermal weight loss from 121 to 392 ◦C of 1.3% (ΔT2) is followed by a fully exothermic one from 392 to 632 ◦C (ΔT3). Overall, P25 under air showed 1.8% weight loss [16].

Comparing the thermal behavior of T3-, T5-, and T7-NPs with commercial P25, also the manufactured NMs, even if calcined, show a weight loss from 35 to 125°C, ascribed to the loss of water molecules adsorbed during air exposure at room environment after calcination, due to the high specific surface of the synthesized nanomaterials. However, in these specimens, the total weight loss was significantly higher than in P25: about 18%, 14%, 3%, respectively, regardless under N_2 or air; this is due to the higher content of carbon-based compounds in the manufactured NMs, which decomposed below 750°C. Indeed, the total weight loss decreased with the increase of calcination temperature; moreover, as expected, only for specimens calcined at T = 300 °C and T = 500 °C a peak of maximum decomposition at Tn=600°C appeared. At higher temperatures, with a maximum decomposition peak at Tn = 900°C, also phosphorous-oxygen compounds decomposed, explaining the continued weight loss until 1300°C, as reported previously [17].

Under N_2, the further weight loss at high temperatures may also be assigned to high temperature TiO_x reduction into TiC due to pyrolyzed carbon, as reported previously [16].

In all prepared PNC-doped-TiO_2-NPs the endothermal transition of TiO_2-NPs from anatase to rutile at T > 500 °C [11,14,15] was not observed, since crystallographic and morphological studies did not detect the phase transformation, even upon 700 °C calcination, which further indicates that the non-metal dopants hinder the phase transformation.

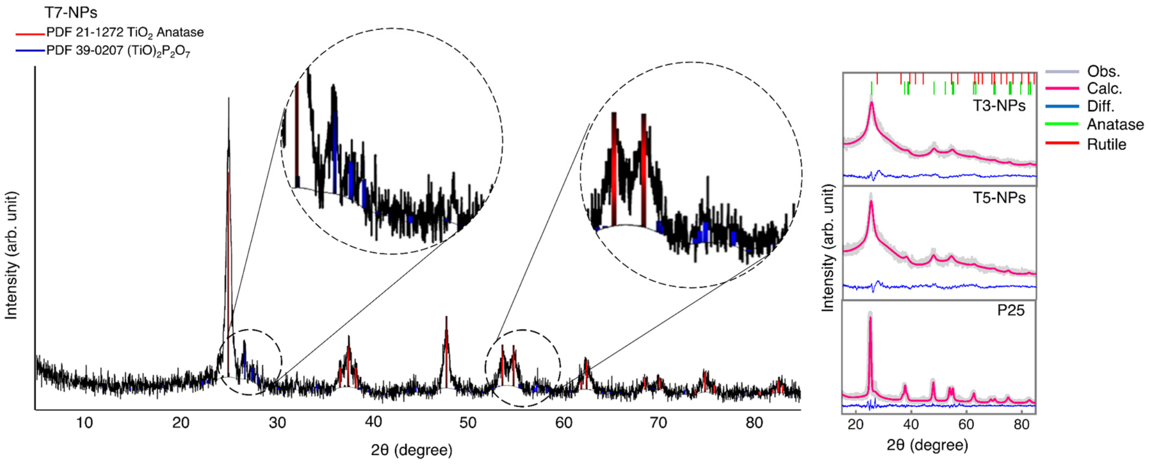

Supporting Fig. S1. XRD diffraction pattern (raw data) of T7-NPs and Rietveld refinement of T3-NPs, T5-NPs and P25.

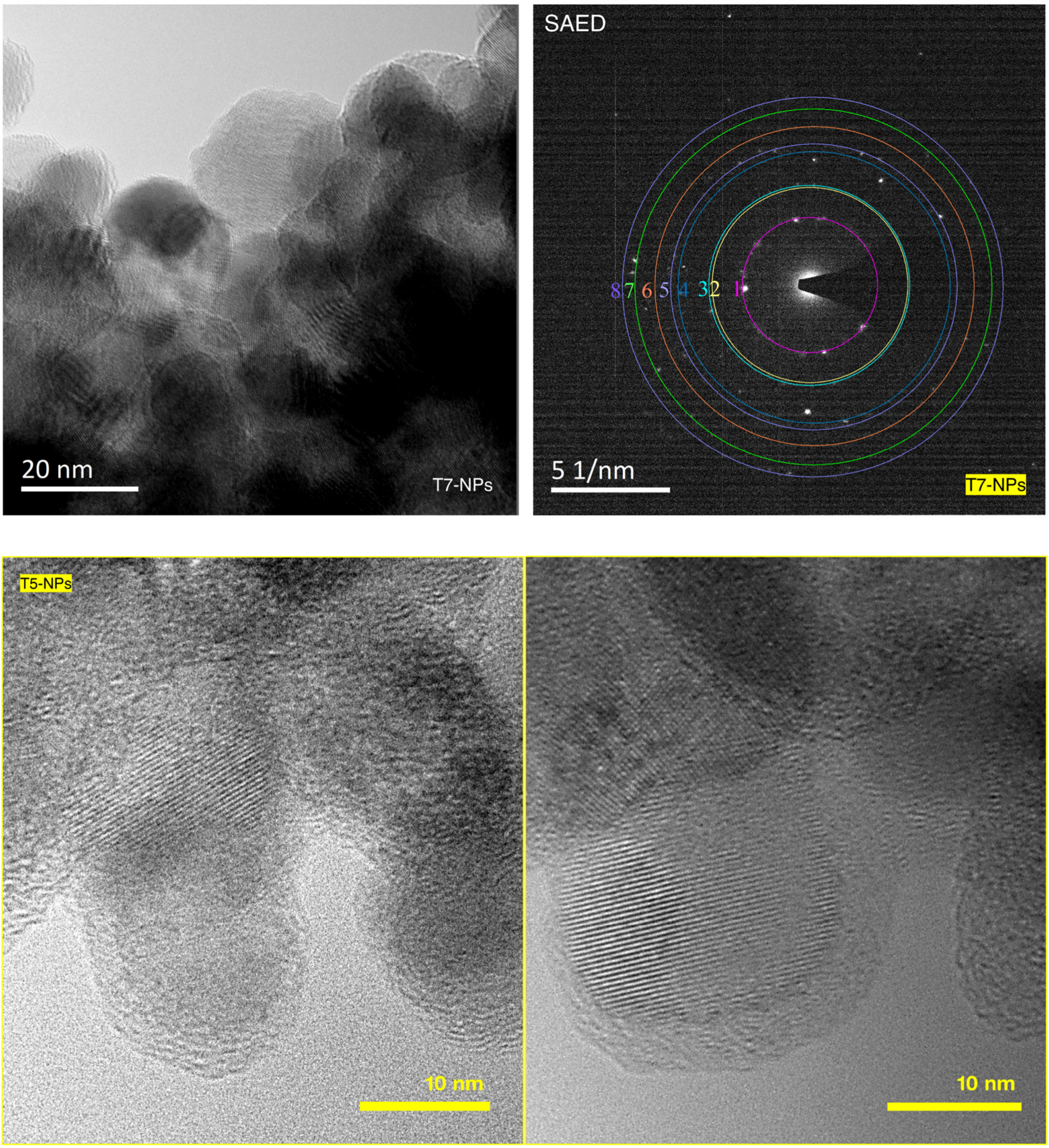

Supporting Fig. S2. Bright-field TEM (top left) with corresponding SAED of T7-NPs. The color circles are a guide for the eye. HRTEM (bottom panels) showing T5-NPs.

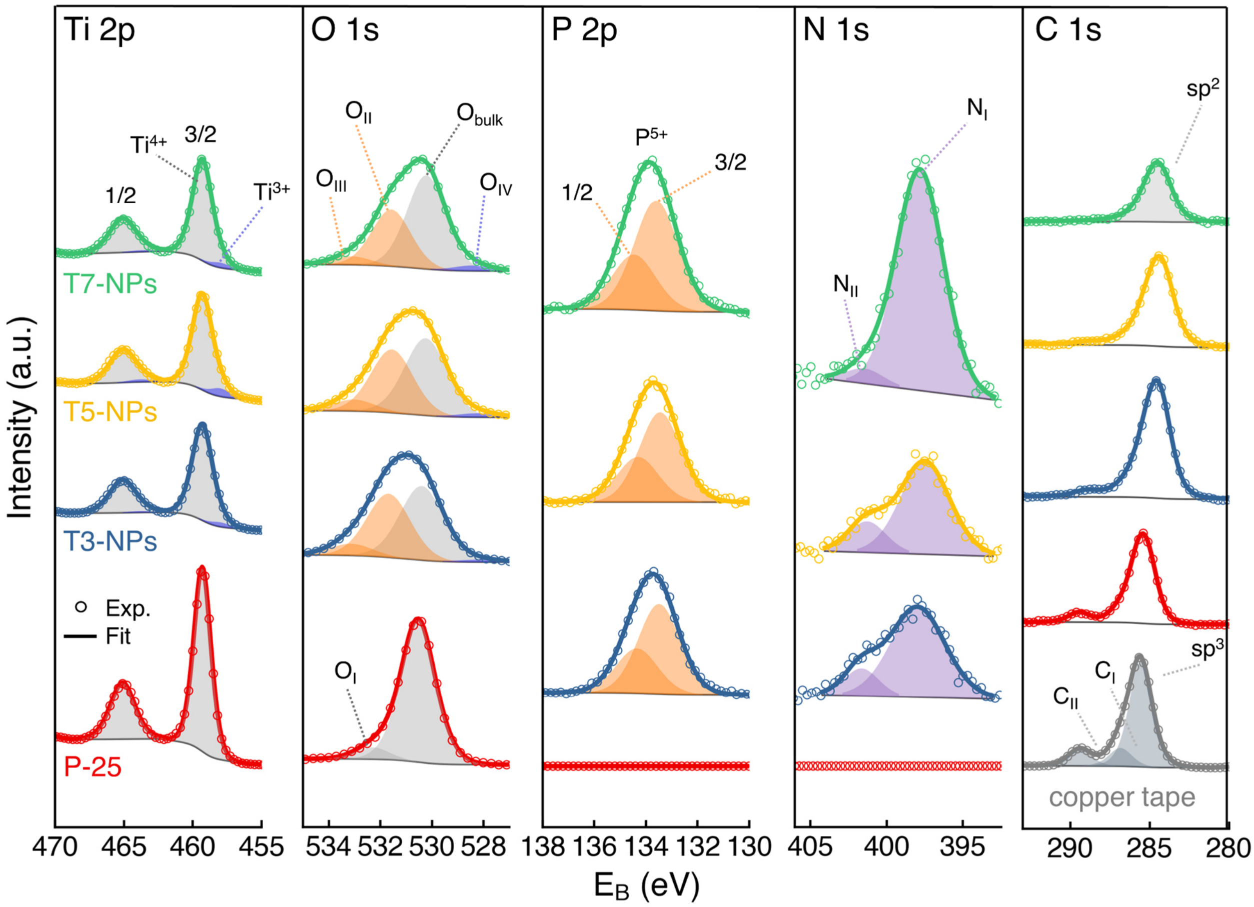

Supporting Fig. S3. XPS of T3-NPs, T5-NPs, T7-NPs and P25.

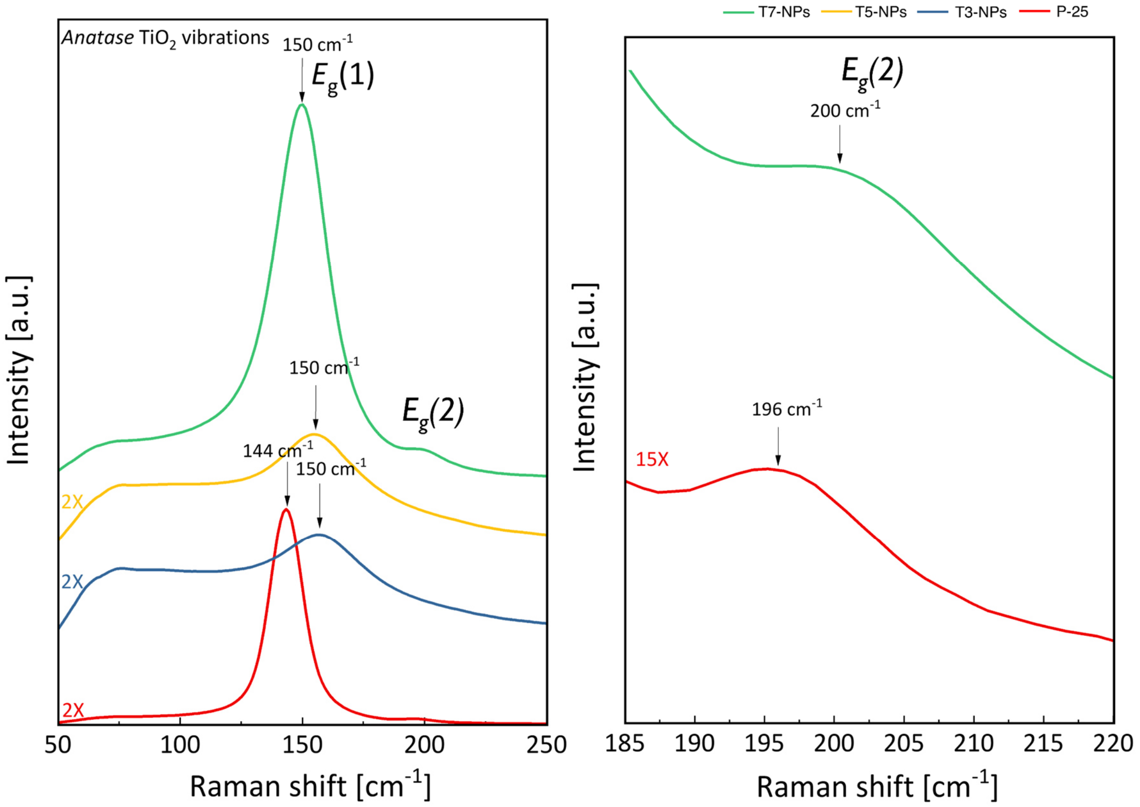

Supporting Fig. S4. Raman vibrations of anatase titania. E_g(1) and (2) vibrations for T3-NPs, T5-NPs, T7-NPs and P25, with magnified E_g(2) for T7-NPs and P25.

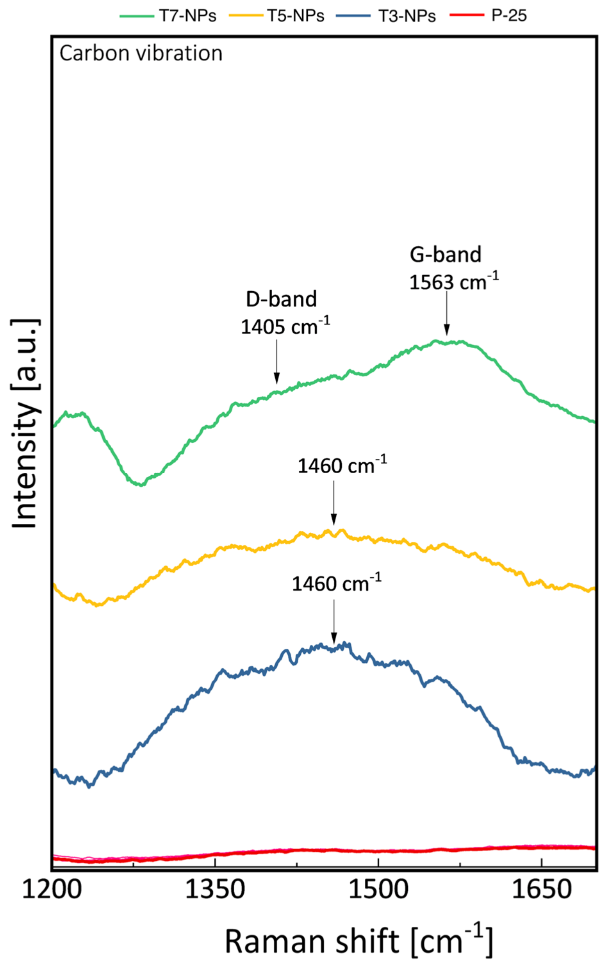

Supporting Fig. S5. Raman spectra of carbon vibrations.

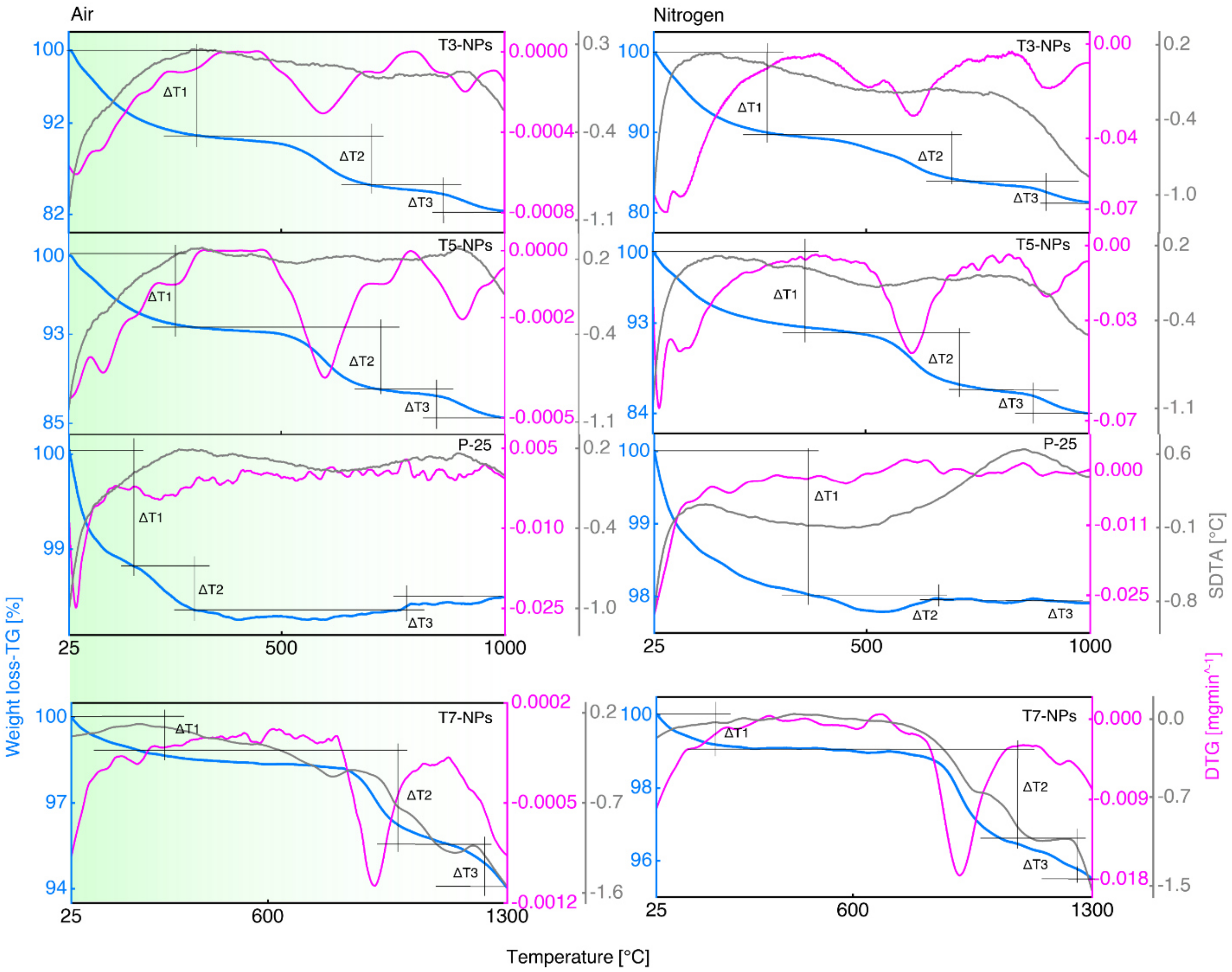

Supporting Fig. S6. TG/DTG/SDTA in air and N_2 of T3-NPs, T5-NPs, T7-NPs and P25.

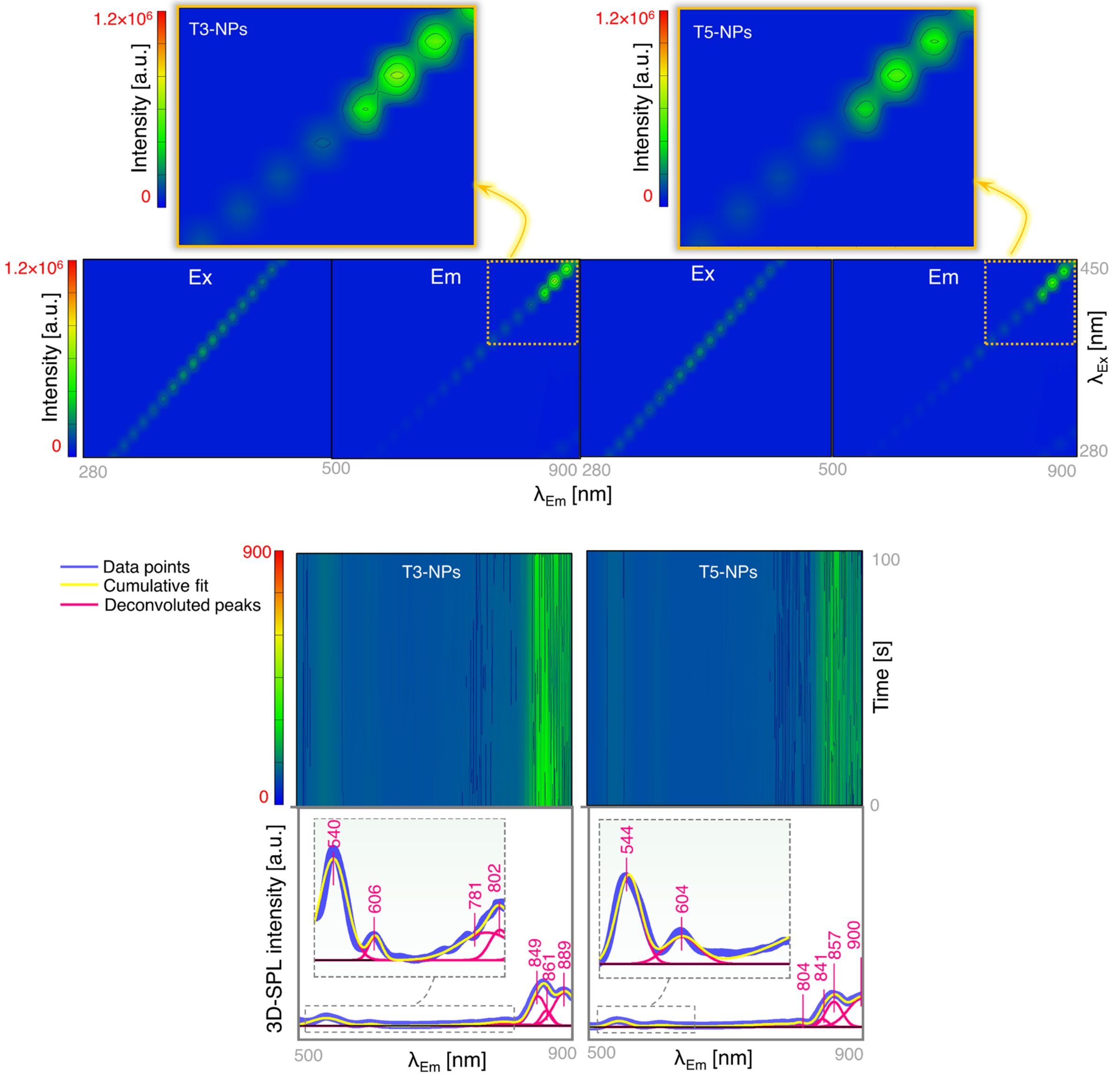

Supporting Fig. S7. (top) 3D photoluminescence (PL) spectroscopy, showing excitation-emission spectrum contour plots of T3-NPs and T5-NPs. (bottom) Synchronous PL (SPL) spectroscopy of T3-NPs and T5-NPs with a fixed wavelength difference of 200 nm ($\Delta\lambda = \lambda_{Em} - \lambda_{Ex} = 200$ nm), λ_{Ex} starting from 300 nm and λ_{Em} from 500 – 900 nm.

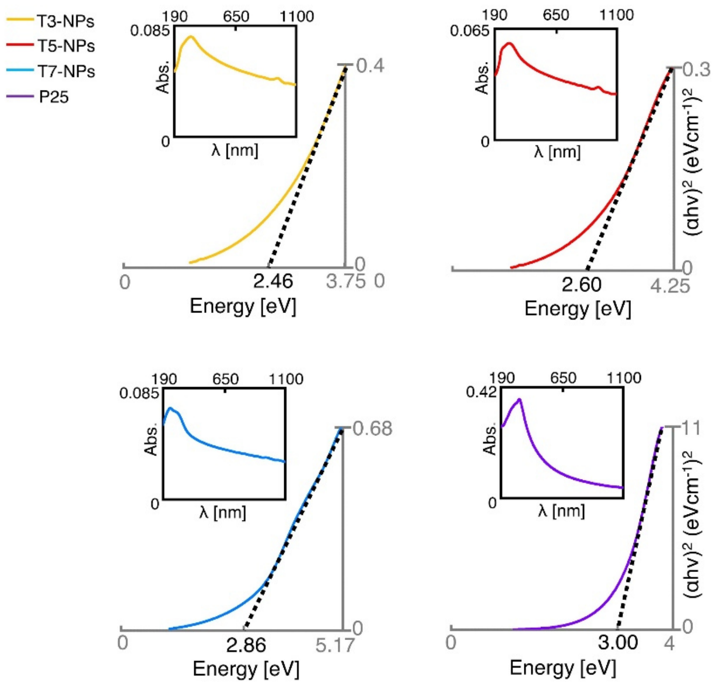

Supporting Fig. S8. Band gap energy estimation (Tauc plot) of T3-NPs, T5-NPs, T7-NPs and P25 (inset: UV-Vis absorption spectra of each specimen).

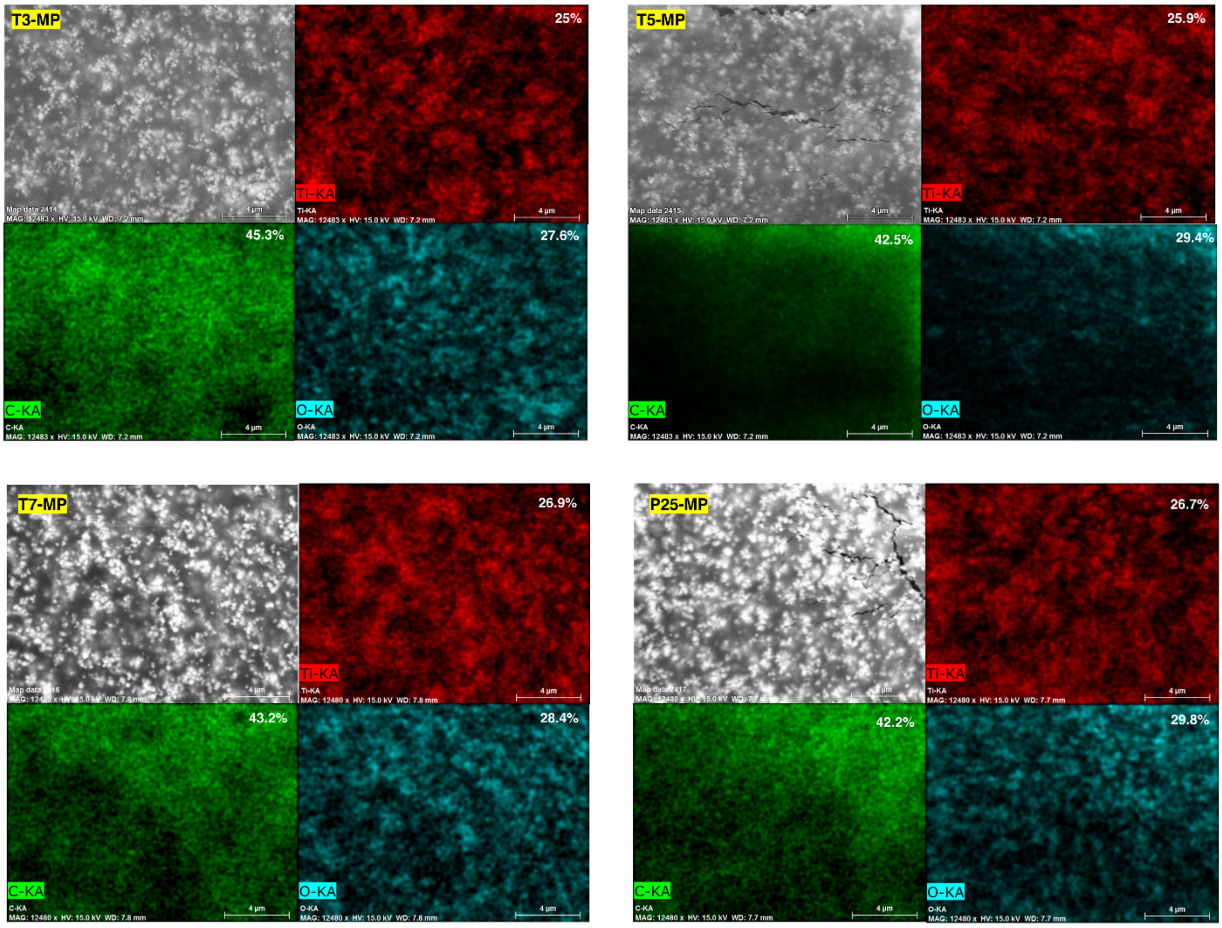

Supporting Fig. S9. SEM and corresponding EDXS mapping showing elemental distribution of TiO_2-NPs (T3-NPs, T5-NPs, T7-NPs, and P25) in paint formulation.

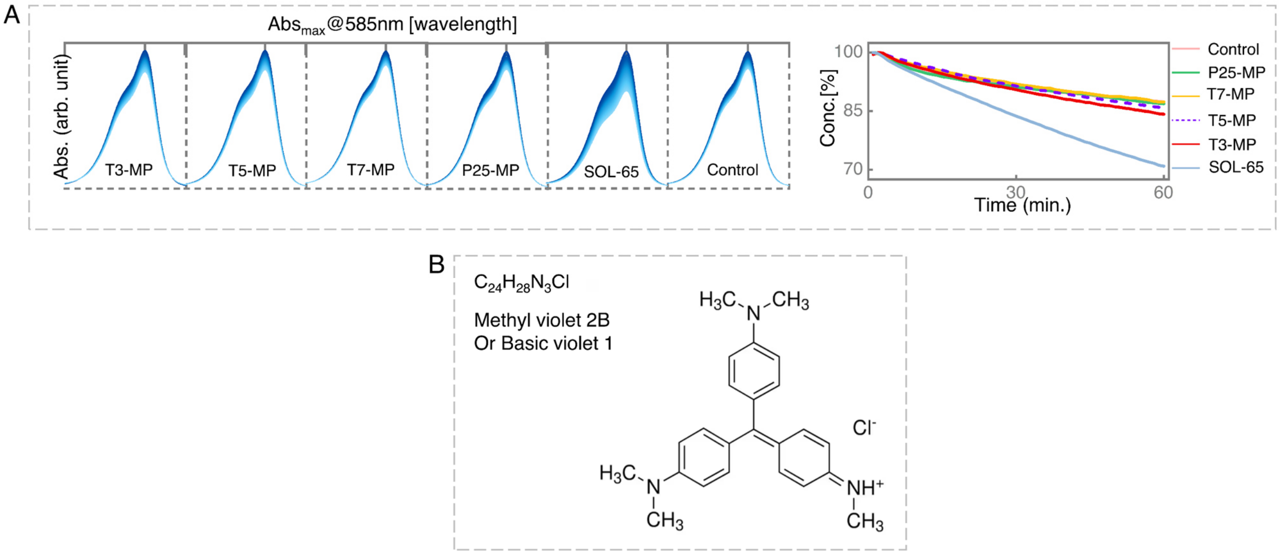

Supporting Fig. S10. (A) Adsorption (in second cycle) of Methyl violet 2B (MV) over the prepared MPs monitored over time through customized *online* UV-Vis spectroscopy. (B) Chemical structure of MV (see main text for details).

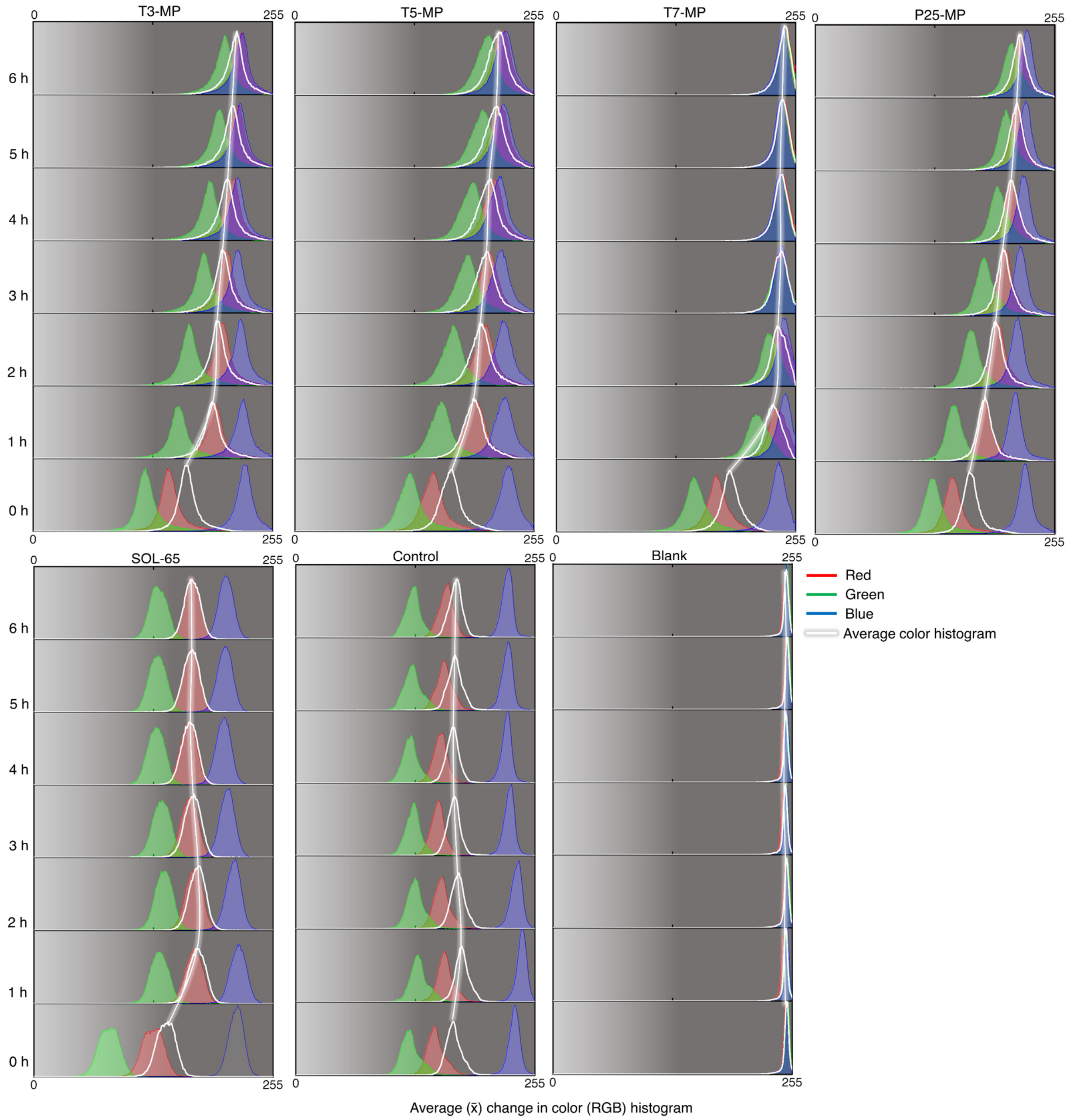

Supporting Fig. S11. Change in RGB (Red, Green, Blue) and average ($\bar{x}$) RGB color histogram values due to photocatalysis in terms of discoloration over time over the surface of specimens described in Fig. 4 (see main text for details).

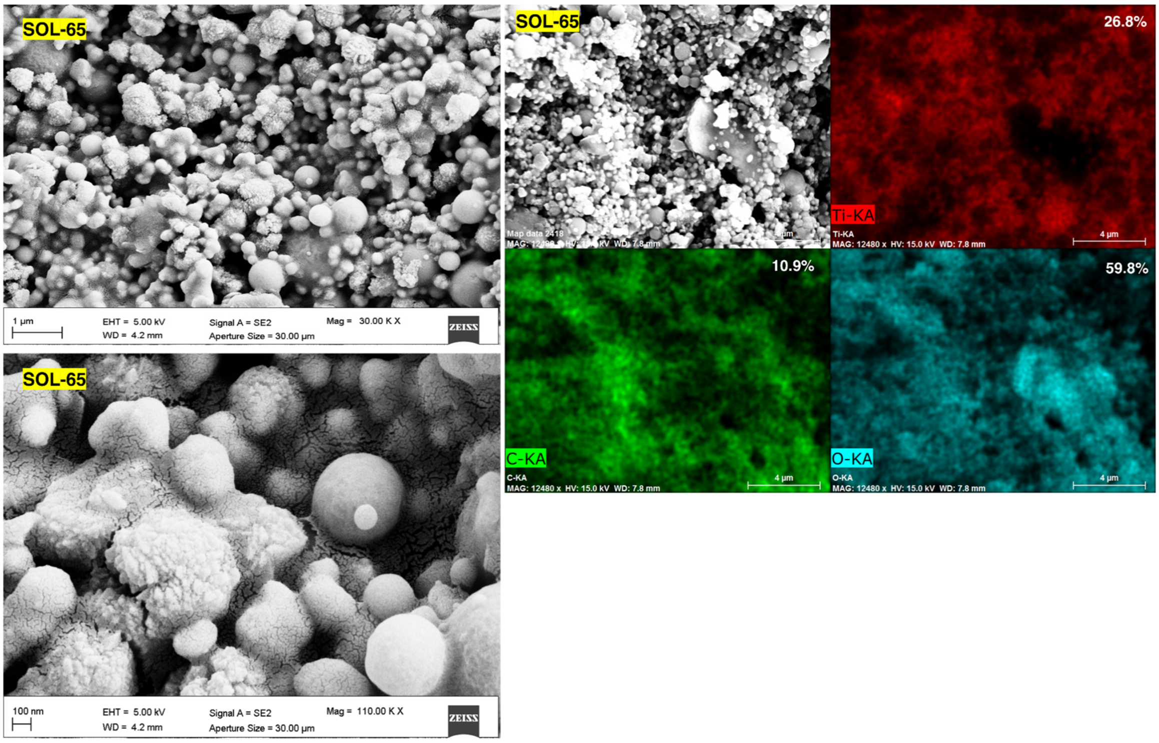

Supporting Fig. S12. SEM and corresponding EDXS mapping showing elemental distribution of SOL-65 (commercial paint).

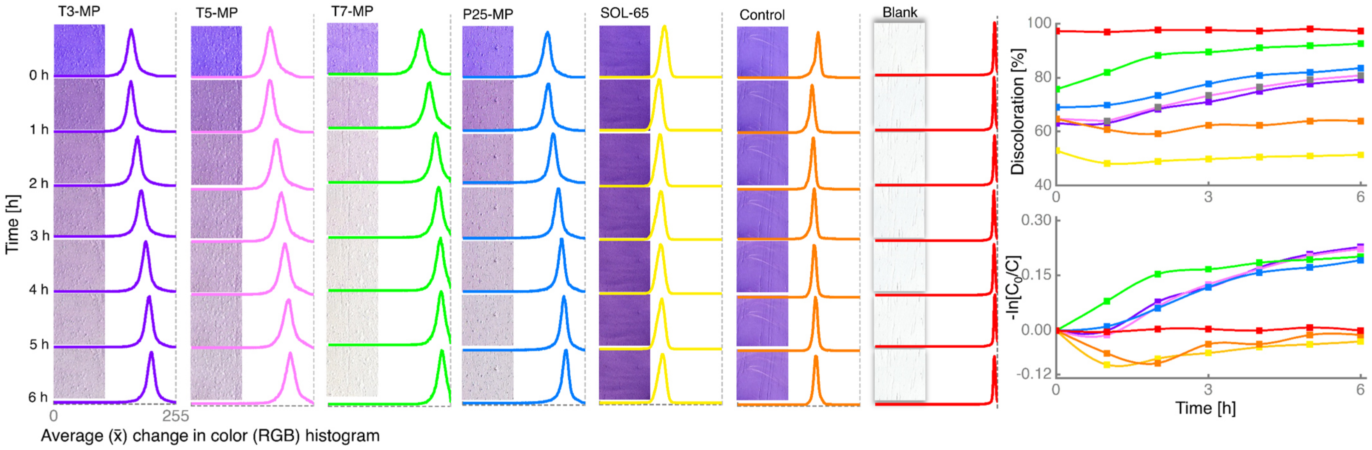

Supporting Fig. S13. (left) Photocatalytic performance (rate of discoloration) in the second cycle of all specimens in natural sunlight (vis. light int.= 465-480 W/m^2, UV light int. = 6.1-6.4 W/m^2). Discoloration (due to photocatalysis) in terms of average (x̄) RGB color histogram change over time. (right) Discoloration percentage over time [h] and -*In* of the measurements.

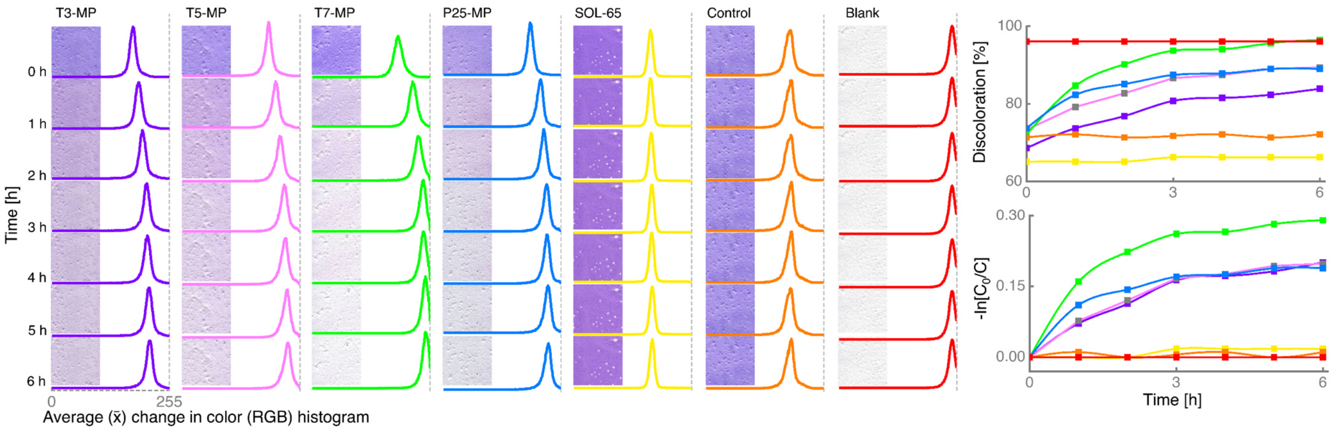

Supporting Fig. S14. (left) Photocatalytic performance (rate of discoloration) of all specimens under UV (UV light int. = 15 W/m^2). Discoloration (due to photocatalysis) in terms of average ($\bar{x}$) RGB color histogram change over time. (right) Discoloration percentage over time [h] and -*In* of the measurements.

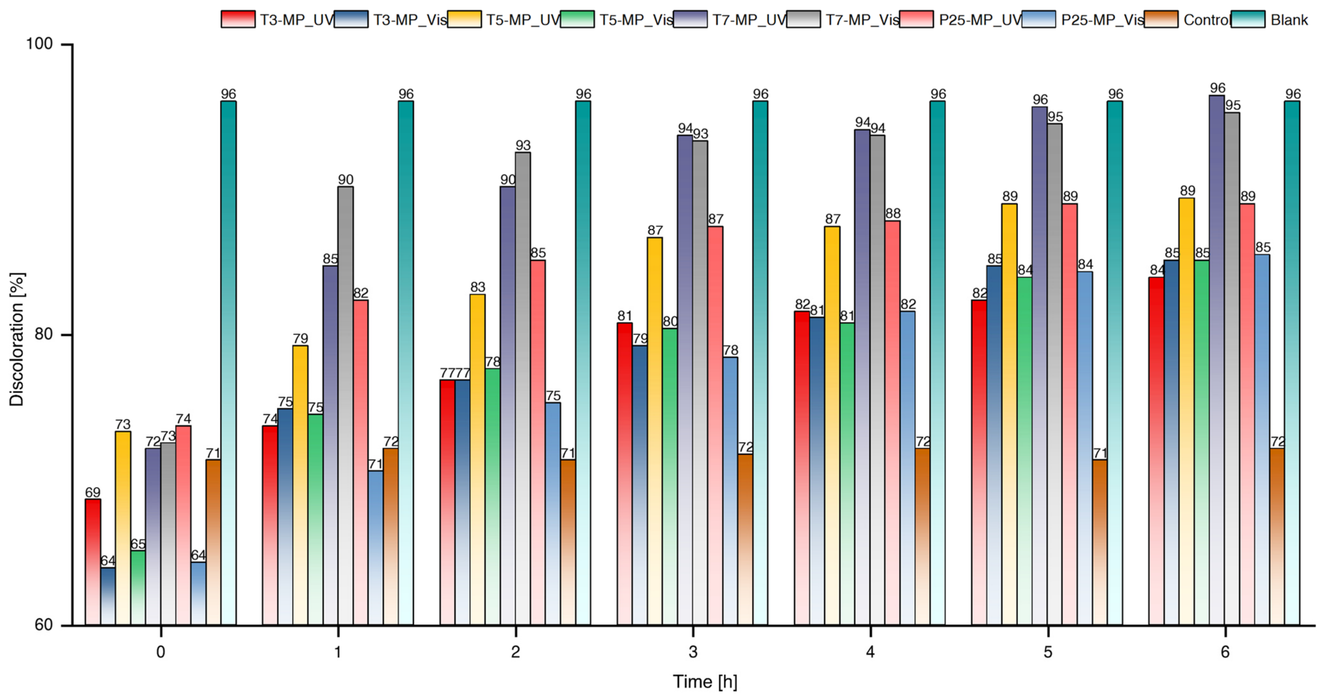

Supporting Fig. S15. Comparison of photocatalysis of T3-MPs, T5-MP, T7-MP and P25-MP under natural sunlight and UV light at room temperature (see main text for details).

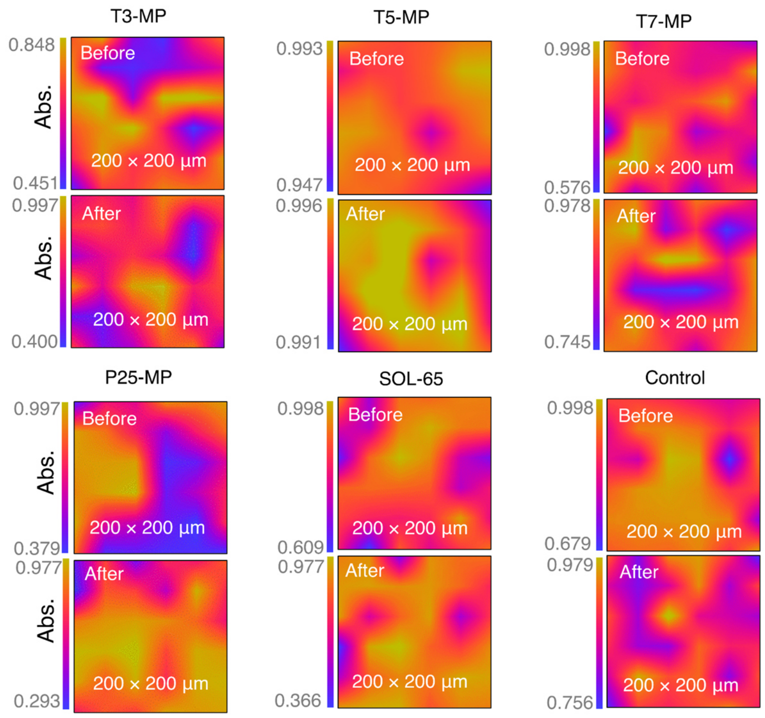

Supporting Fig. S16. Topographic distribution (correlation map) of T3-MP, T5-MP, T7-MP, P25-MP and SOL-65, before and after photoacatalysis (see main text for details).

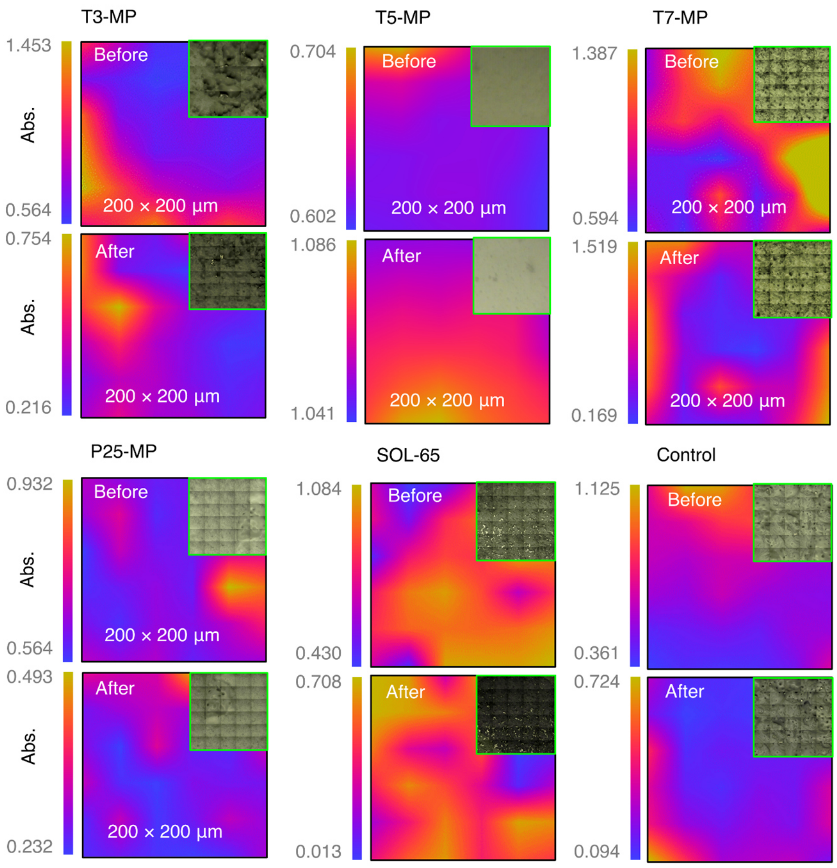

Supporting Fig. S17. Topographic distribution (energy map) and corresponding micrograph (inset) of T3-MP, T5-MP, T7-MP, P25-MP and SOL-65, before and after photoacatalysis (see main text for details).

Supporting Table S1. Crystal parameters of T3-NPs, T5-NPs, T7-NPs and P25.

FWHM	Peak pos. [°2Th]	Theta	Theta in Radians	d in Radians	Crystallite size [Å]	Crystallite size [nm]	d-Spacing [Å]	d-Spacing [nm]	Average crystallite size [nm]
					**T3-NPs**				
3.95	25.50	12.75	0.22	0.06	20.59	2.05	3.48	0.34	3.38
10.40	36.67	18.33	0.32	0.18	8.041	0.80	2.44	0.24	
2.34	47.99	23.99	0.41	0.04	37.02	3.70	1.89	0.18	
2.18	54.44	27.22	0.47	0.03	40.89	4.08	1.68	0.16	
0.73	62.29	31.14	0.54	0.01	126.53	12.65	1.48	0.14	
4.49	68.99	34.49	0.60	0.07	21.43	2.14	1.36	0.13	
11.39	74.24	37.12	0.64	0.19	8.74	0.87	1.27	0.12	
14.31	82.20	41.10	0.71	0.24	7.36	0.73	1.17	0.11	
					**T5-NPs**				
3.22	25.34	12.67	0.22	0.05	25.26	2.52	3.511	0.35	2.10
8.62	37.57	18.78	0.32	0.15	9.72	0.97	2.39	0.239	
1.96	47.93	23.96	0.41	0.03	44.20	4.42	1.89	0.18	
2.30	54.41	27.20	0.47	0.04	38.81	3.88	1.68	0.16	
7.51	61.60	30.80	0.53	0.13	12.30	1.23	1.50	0.15	
14.83	68.24	34.12	0.59	0.25	6.46	0.64	1.37	0.13	
3.92	74.60	37.30	0.65	0.06	25.42	2.54	1.27	0.12	
17.62	81.53	40.76	0.71	0.30	5.95	0.59	1.17	0.11	
					**T7-NPs**				
0.52	25.33	12.66	0.22	0.01	155.31	15.53	3.51	0.35	9.63
0.59	26.95	13.48	0.24	0.01	138.84	13.88	3.31	0.33	
0.38	32.44	16.22	0.28	0.01	215.28	21.53	2.76	0.28	
1.51	37.80	18.90	0.33	0.03	55.43	5.54	2.38	0.24	
1.05	44.82	22.41	0.39	0.02	81.47	8.15	2.02	0.20	
0.73	48.05	24.03	0.42	0.01	119.58	11.96	1.89	0.19	

3.06	54.53	27.26	0.48	0.05	29.24	2.92	1.68	0.17	
1.13	62.65	31.33	0.55	0.02	82.03	8.20	1.48	0.15	
11.36	69.44	34.72	0.61	0.20	8.51	0.85	1.35	0.14	
1.41	75.10	37.55	0.66	0.02	71.09	7.11	1.26	0.13	
1.04	82.85	41.42	0.72	0.02	102.11	10.21	1.16	0.12	

P25

0.43	25.21	12.60	0.22	0.01	190.18	19.02	3.53	0.35	
0.39	27.33	13.67	0.24	0.01	209.58	20.96	3.26	0.33	
0.47	36.00	18.00	0.31	0.01	177.16	17.72	2.49	0.25	
0.83	36.91	18.46	0.32	0.01	101.16	10.12	2.43	0.24	
0.53	37.73	18.86	0.33	0.01	157.55	15.76	2.38	0.24	
0.89	38.36	19.18	0.33	0.02	94.16	9.42	2.34	0.23	
0.48	41.14	20.57	0.36	0.01	178.21	17.82	2.19	0.22	
0.44	43.92	21.96	0.38	0.01	196.46	19.65	2.06	0.21	
0.53	47.93	23.97	0.42	0.01	163.42	16.34	1.90	0.19	14.33
0.84	53.96	26.98	0.47	0.01	106.11	10.61	1.70	0.17	
0.71	54.93	27.46	0.48	0.01	126.33	12.63	1.67	0.17	
0.57	56.48	28.24	0.49	0.01	157.13	15.71	1.63	0.16	
0.74	62.58	31.29	0.55	0.01	126.09	12.61	1.48	0.15	
0.67	68.78	34.39	0.60	0.01	143.79	14.38	1.36	0.14	
0.86	70.12	35.06	0.61	0.02	112.64	11.26	1.34	0.13	
0.69	75.00	37.50	0.65	0.01	145.51	14.55	1.27	0.13	
1.05	75.86	37.93	0.66	0.02	95.72	9.57	1.25	0.13	
1.09	82.64	41.32	0.72	0.02	97.44	9.74	1.17	0.12	

Supporting Table S2. SAED analysis of T7-NPs with their corresponding phase identifiers.

Substance ID	5531913316051761806	**No.**	**h**	**k**	**l**	**d nm**
Material	TiO_2	1	1	0	1	0.35
Crystal system	tetragonal	2	1	0	3	0.24
Space group	$I4_1/amd$	3	0	0	4	0.23
Space group number	(141)	4	2	0	2	0.17
Phase	Anatase	5	2	1	1	0.16
		6	2	1	3	0.15
		7	2	2	0	0.13
		8	3	0	1	0.125

Supporting Table S3. Elemental composition of T3-NPs, T5-NPs, T7-NPs and P25 determined by XPS.

**Method**	**XPS**				
**Sample**	**c_{Ti} /at-%**	**c_P /at-%**	**c_N /at-%**	**c_O /at-%**	**c_C /at-%**
T3-NPs	11.5	7.1	2.6	54.4	24.3
T5-NPs	12.4	7.4	2.7	57.3	20.2
T7-NPs	13.1	10.0	5.4	58.4	13.2
P25	22.4	0	0	57.0	20.6

Supporting Table S4. Thermal properties (main temperature intervals ΔTn, corresponding weight loss, temperatures of maximum decomposition (in brackets), if observed, and total weight loss percentage of each sample in nitrogen or air.

**Sample**		**Nitrogen**				**Air**			
		**ΔT1**	**ΔT2**	**ΔT3**	**Total**	**ΔT1**	**ΔT2**	**ΔT3**	**Total**
T3-NPs	T (°C)	40-300 (90)	300-800 (600)	800-1000 (900)	17.6	40-300 (90)	300-800	800-1000 (600)	16.8
	- Δm (%)	9.1	6.3	2.2		8.4	6.1	2.3	
T5-NPs	T (°C)	40-350 (90)	350-800 (600)	800-1000 (900)	15.2	40-350 (90)	350-800 (600)	800-1000 (900)	13.9
	- Δm (%)	6.5	6.4	2.2		5.9	5.7	2.2	
T7-NPs	T (°C)	40-700	700-1000-	1000-1300-	4.3	40-700	700-1000 (900)	1000-1300	5.7
	- Δm (%)	0.9	2.2	1.3		1.4	2.2	1.3	
P25	T (°C)	35-125 (40)	125-266 (180)	267-568	1.4	33-121 (40)	121-392 (220)	392-632	1.8
	- Δm (%)	0.6	0.5	0.3		0.7	1.3	0.16	

Supporting Table S5. Comparison of photocatalytic performance of PNC-doped anatase TiO_2-NPs as photocatalytic additive to paint with TiO_2-based surface coating materials reported in literature.

Synthesis method	Synthesis mechanism	Material (final product)	Pollutant	Light source	Degradation efficiency (1 h)	Photocatalytic mechanism	Ref
Sustainable nanosynthesis	ATR-FTIR	2.5% PNC doped anatase-TiO_2 nanoparticles in water-based paint<br>(Coating)	*Methyl violet	UV/Vis. 15W/m^2<br>465 W/m^2 (solar light)	≈ 90%	3D-synchronous photoluminescence spectroscopy (3D-SPL) supported by X-ray photoelectron spectroscopy (XPS)	This work
Screen-printing	-	TiO_2 over glazed ceramic tiles<br>(Coating)	Orange-II	Vis. Philips ML-160 W lamp	≤ 50%<br>5 (55×136 μm) coated tiles VS 20 mg/L of P)	-	18
Electrospinning	-	Ag-TiO_2/nylon-6 nanocomposite<br>(Mat)	Methylene blue	UV/Vis. Solar radiation 16.21 MJ/m^2	≈ 60%<br>(4 cm×4 cm mat VS 25mL of 10 ppm of P)	-	19
	-	ZnO/C-TiO_2 core-sheath<br>(Nanofiber film)	Rhodamine-B	UV/Vis. 500 W Hg lamp	≈ 85%<br>(60 mg VS 60 mL of 10mg/L of P)	-	20
MAPLE<br>(Matrix-assisted pulsed laser evaporation)	-	TiO_2/N-doped RGO<br>TiO_2/N-doped RGO/g-C_3N_4<br>(Thin layer)	Chloramphenicol and Methyl orange	UV 8W×16 lamps	≈ 55%<br>(As prepared sample VS 4 ppm of P)	Radical scavenging test (Ascorbic acid and tert-butyl-alcohol)	21

Hydrothermal method	-	TiO_2 over bauxite-based hollow fiber<br>(Membrane)	Bisphenol-A	UV/Vis. 36W	≈ 20%<br>(10–15 cm membrane VS 50 ppm of P)	-	22
Pulsed laser co-deposition	-	Au nanoparticles-TiO_2 nanostructures, over silica substrate<br>(Thin film)	Methyl orange	UV/Vis. 550W, Abet tech.	≈ 60%<br>(Thin film VS 2.5 mL of a 10^{-5} M of P)	-	23
Cold atmospheric plasma	-	N-doped TiO_2<br>(Coating)	Methylene blue	Vis. 10 W, Philip	< 20%<br>(10 cm^2 film VS 30mL of 5 mg/L of P)	XPS and Raman spectroscopy	24
Atomic layer deposition	-	TiO_2 over Kapton (polymer)<br>(Thin film)	*4-*chlorophenol	UV 300 W Xe arc lamp (Oriel)	≈ 20%<br>(Thin film VS 0.1 mM of P)	-	25
Dip coating	-	C-N-TiO_2 over stainless steel<br>(Coating)	Orange II sodium	UV/Vis. 100 mW/cm^2 solar light<br>160W-UV lamp	≈50%<br>(6 cm×2 cm meshes VS 500 mL of 5mg/L of P)	Raman spectroscopy	26
Spray coating	-	TiO_2 in *n*-heptane over stone<br>(Coating)	Methyl red	UV/Vis. 150W, Xe lamp	< 10%<br>($5 \times 1 \times 2 cm^3$ substrate VS 9.4×10^{-9} $mg \cdot cm^{-2}$ of P)	PL	27

Plasma spray coating	-	TiO_2 nanoparticles in ethanol (mixed with PEG and PVP) over stainless plates (Coating)	Methyl blue	UV 15W, Xe lamp	< 20% (20×20×2 mm substrate VS 15 mL of 5ppm of P)	-	28

*Methyl violet 2B (also called Basic Violet 1 or simply termed Methyl Violet)

Supporting Table S6. Summary of results obtained from different physicochemical characterization methods.

**Specimen**	**Methods with summarized results**							
	**ATR-FTIR**	**DRIFTS**	**XRD**	**XPS**	**Raman spectroscopy**	**EFTEM /EELS**	**3D-PL spectroscopy**	**UV spectroscopy**
**Objective**	Synthesis mechanism	Surface chemistry	Phase	Main elements and oxidation states	Surface chemistry	High resolution elemental microscopy	(e^{-}/h^{+}) recombination analysis	Bandgap energy (eV)
T3-NPs	$Ti_3(PO_4)_4.xH_2O$ reduction into PNC doped TiO_2-NPs<br><br>Metabolites from fallen-leaves extract involved,<br><br>i. ν C-O (carboxylic acid, ether, alcoholic, and carbonyl groups).<br>ii. ν C-OH (flavonoids)<br>iii. ν C-N (aromatic amines, alkaloids)<br>iv. ν $C-H_2$ (flavonoids)<br>v. ν CH_2 and ν CH_3 (fatty acid -acyl group)	i. ν P-O-C, ν P-O-P deformation vibrations, and ν P=O<br>ii. ν N-H, and ν C=N (imine group)<br>iii. ν C-C<br>iv. Anatase<br>v. H-O-H	Anatase	Ti^{4+}(TiO_2)<br>Ti^{3+}<br><br>O^{2-}<br>(TiO_2, $P_xO_y^{z-}$)<br><br>P^{5+}<br>(PO_4^{3-}, $P_2O_7^{4-}$)<br><br>N<br>C=N/C≡N	ν P-O<br>ν C=N<br>Amorphous C<br>ν C-C<br>Anatase	Ti L-edges including L_3 and L_2 (457, 462 eV),<br><br>O k-edge (~534 eV),<br><br>P L-edge (~162 eV),<br><br>N K-edge (~407 eV)<br><br>and C k-edge (~303 eV)	For PNC doped TiO_2-NPs, e^{-}/h^{+} recombination and relaxation occur at, valance band (VB) to the conduction band (CB), as well as interference with shallow trap states (STS) beneath the CB, connected with oxygen vacancies (OV), surface oxygen vacancies (SOV), structural defects (SD), impurities, and unbalanced Ti atoms on the surface.<br><br>PNC doped TiO_2-NPs are 7.3-times stronger inhibitor of photoinduced e–/h+ recombination than P25	2.46 eV
T5-NPs		i. ν P-O-C, ν P-O-P deformation vibrations, and ν P=O<br>ii. ν N-H, and ν C=N (imine group)<br>iii. ν C-C<br>iv. Anatase<br>v. H-O-H	Anatase	Ti^{4+}(TiO_2)<br>Ti^{3+}<br><br>O^{2-}<br>(TiO_2, $P_xO_y^{z-}$)<br><br>P^{5+}<br>(PO_4^{3-}, $P_2O_7^{4-}$)<br><br>N<br>C=N/C≡N	ν P-O<br>ν C=N<br>Amorphous C<br>ν C-C<br>Anatase			2.60 eV
T7-NPs		i. ν P-O-C, ν P-O-P deformation vibrations, and ν P=O<br>ii. ν N-H, and ν C=N (imine and nitrile group)<br>iii. ν C-C<br>iv. Anatase<br>v. H-O-H	Anatase	Ti^{4+}(TiO_2)<br>Ti^{3+}<br><br>O^{2-}<br>(TiO_2, $P_xO_y^{z-}$)<br><br>P^{5+}<br>(PO_4^{3-}, $P_2O_7^{4-}$)<br><br>N<br>C=N/C≡N	ν P-O-P<br>ν P-O<br>ν C=N<br>Amorphous C<br>Mutated graphite<br>ν C-C<br>Anatase			2.68 eV
P25	Commercial specimen<br>N/A*	i. Anatase /rutile<br>ii. H-O-H	Anatase<br>Rutile	Ti^{4+}<br>(TiO_2)<br>O^{2-}<br>(TiO_2)	Anatase<br>Rutile	N/A*		3.00 eV

*N/A information not available

Supporting References

(1) Maqbool, Q.; Barucca, G.; Sabbatini, S.; Parlapiano, M.; Ruello, M. L.; Tittarelli, F. Transformation of Industrial and Organic Waste into Titanium Doped Activated Carbon – Cellulose Nanocomposite for Rapid Removal of Organic Pollutants. *J. Hazard. Mater.* **2022**, *423*, 126958. https://doi.org/10.1016/J.JHAZMAT.2021.126958.

(2) Maqbool, Q.; Nazar, M.; Naz, S.; Hussain, T.; Jabeen, N.; Kausar, R.; Anwaar, S.; Abbas, F.; Jan, T. Antimicrobial Potential of Green Synthesized CeO2 Nanoparticles from Olea Europaea Leaf Extract. *Int. J. Nanomedicine* **2016**, *11*, 5015–5025. https://doi.org/10.2147/IJN.S113508.

(3) Gao, L.; Rao, B.; Dai, H.; Xie, H.; Wang, P.; Ma, F. Kinetics of Sulphuric Acid Leaching of Titanium from Refractory Anatase under Atmospheric Pressure. *Physicochem. Probl. Miner. Process.* **2019**. https://doi.org/10.5277/ppmp18159.

(4) Devilliers, D.; Dinh, M. T.; Mahé, E.; Krulic, D.; Larabi, N.; Fatouros, N. Behaviour of Titanium in Sulphuric Acid - Application to DSAs. *J. New Mater. Electrochem. Syst.* **2006**.

(5) Xu, Y.; Yamazaki, M.; Villars, P. Inorganic Materials Database for Exploring the Nature of Material. *Jpn. J. Appl. Phys.* **2011**, *50* (11 PART 2), 11RH02. https://doi.org/10.1143/JJAP.50.11RH02/XML.

(6) Maqbool, Q.; Yigit, N.; Stöger-Pollach, M.; Ruello, M. L.; Tittarelli, F.; Rupprechter, G. Operando Monitoring of a Room Temperature Nanocomposite Methanol Sensor. *Catal. Sci. Technol.* **2023**, *13* (3), 624–636. https://doi.org/10.1039/D2CY01395A.

(7) Diebold, U.; Madey, T. E. TiO2 by XPS. *Surf. Sci. Spectra* **2021**, *4* (3), 227. https://doi.org/10.1116/1.1247794.

(8) Global Solar Atlas https://globalsolaratlas.info/detail?c=43.571437,13.355942,10&s=43.58659,13.515992&m=site (accessed 2022 -10 -07).

(9) Conti, C.; Ferraris, P.; Garavaglia, M.; Giorgini, E.; Rubini, C.; Sabbatini, S.; Tosi, G. Microimaging FTIR of Head and Neck Tumors. IV. *Microsc. Res. Tech.* **2009**. https://doi.org/10.1002/jemt.20644.

(10) Blume, R.; Rosenthal, D.; Tessonnier, J. P.; Li, H.; Knop-Gericke, A.; Schlögl, R. Characterizing Graphitic Carbon with X-Ray Photoelectron Spectroscopy: A Step-by-Step Approach. *ChemCatChem* **2015**, *7* (18), 2871–2881. https://doi.org/10.1002/CCTC.201500344.

(11) Mahlambi, M. M.; Mishra, A. K.; Mishra, S. B.; Krause, R. W.; Mamba, B. B.; Raichur, A. M. Comparison of Rhodamine B Degradation under UV Irradiation by Two Phases of Titania Nano-Photocatalyst. *J. Therm. Anal. Calorim.* **2012**, *110* (2), 847–855. https://doi.org/10.1007/S10973-011-1852-7/FIGURES/10.

(12) Li, X. W.; Song, R. G.; Jiang, Y.; Wang, C.; Jiang, D. Surface Modification of TiO2 Nanoparticles and Its Effect on the Properties of Fluoropolymer/TiO2 Nanocomposite Coatings. *Appl. Surf. Sci.* **2013**, *276*, 761–768. https://doi.org/10.1016/J.APSUSC.2013.03.167.

(13) Spada, E. R.; Pereira, E. A.; Montanhera, M. A.; Morais, L. H.; Freitas, R. G.; Costa, R. G. F.; Soares, G. B.; Ribeiro, C.; de Paula, F. R. Preparation, Characterization and Application of Phase-Pure Anatase and Rutile TiO2 Nanoparticles by New Green Route. *J. Mater. Sci. Mater. Electron.* **2017**, *28* (22), 16932–16938. https://doi.org/10.1007/S10854-017-7613-Z/TABLES/1.

(14) Cerro-Prada, E.; García-Salgado, S.; Quijano, M. Á.; Varela, F. Controlled Synthesis and Microstructural Properties of Sol-Gel TiO2 Nanoparticles for Photocatalytic Cement Composites. *Nanomater. 2019, Vol. 9, Page 26* **2018**, *9* (1), 26. https://doi.org/10.3390/NANO9010026.

(15) Zhu, X.; Han, S.; Feng, W.; Kong, Q.; Dong, Z.; Wang, C.; Lei, J.; Yi, Q. The Effect of Heat Treatment on the Anatase–Rutile Phase Transformation and Photocatalytic Activity of Sn-Doped TiO2 Nanomaterials. *RSC Adv.* **2018**, *8* (26), 14249–14257. https://doi.org/10.1039/C8RA00766G.

(16) Maqbool, Q.; Czerwinska, N.; Giosue, C.; Sabbatini, S.; Ruello, M. L.; Tittarelli, F. New Waste-Derived TiO2 Nanoparticles as a Potential Photocatalytic Additive for Lime Based Indoor Finishings. *J. Clean. Prod.* **2022**, 133853. https://doi.org/10.1016/J.JCLEPRO.2022.133853.

(17) Nalini, V.; Haugsrud, R.; Norby, T. High-Temperature Proton Conductivity and Defect Structure of TiP2O7. *Solid State Ionics* **2010**, *181* (11–12), 510–516. https://doi.org/10.1016/J.SSI.2010.02.017.

(18) Marcos, P. S.; Marto, J.; Trindade, T.; Labrincha, J. A. Screen-Printing of TiO2 Photocatalytic Layers on Glazed Ceramic Tiles. *J. Photochem. Photobiol. A Chem.* **2008**, *197* (2–3), 125–131. https://doi.org/10.1016/J.JPHOTOCHEM.2007.12.017.

(19) Pant, H. R.; Pandeya, D. R.; Nam, K. T.; Baek, W. il; Hong, S. T.; Kim, H. Y. Photocatalytic and Antibacterial Properties of a TiO2/Nylon-6 Electrospun Nanocomposite Mat Containing Silver Nanoparticles. *J. Hazard. Mater.* **2011**, *189* (1–2), 465–471. https://doi.org/10.1016/J.JHAZMAT.2011.02.062.

(20) Song, L.; Ning, L.; Zhai, J.; Guan, Y.; Ke, H.; Jie, X. Preparation of ZnO/Carbon-TiO2 Core-Sheath Nanofibers Film with Enhanced Photocatalytic Properties. *Appl. Phys. A Mater. Sci. Process.* **2020**, *126* (12), 1–7. https://doi.org/10.1007/S00339-020-04136-7/FIGURES/8.

(21) Ivan, R.; Pérez del Pino, A.; Yousef, I.; Logofatu, C.; György, E. Laser Synthesis of TiO2–Carbon Nanomaterial Layers with Enhanced Photodegradation Efficiency towards Antibiotics and Dyes. *J. Photochem. Photobiol. A Chem.* **2020**, *399*, 112616. https://doi.org/10.1016/J.JPHOTOCHEM.2020.112616.

(22) Ismail, N. J.; Othman, M. H. D.; Abu Bakar, S.; Sheikh Abdul Kadir, S. H.; Abd Aziz, M. H.; Pauzan, M. A. B.; Hubadillah, S. K.; El-badawy, T.; Jaafar, J.; A Rahman, M. Hydrothermal Synthesis of TiO2 Nanoflower Deposited on Bauxite Hollow Fibre Membrane for Boosting Photocatalysis of Bisphenol A. *J. Water Process Eng.* **2020**, *37*, 101504. https://doi.org/10.1016/J.JWPE.2020.101504.

(23) Bricchi, B. R.; Ghidelli, M.; Mascaretti, L.; Zapelli, A.; Russo, V.; Casari, C. S.; Terraneo, G.; Alessandri, I.; Ducati, C.; Li Bassi, A. Integration of Plasmonic Au Nanoparticles in TiO2 Hierarchical Structures in a Single-Step Pulsed Laser Co-Deposition. *Mater. Des.* **2018**, *156*, 311–319. https://doi.org/10.1016/J.MATDES.2018.06.051.

(24) Chen, Q.; Ozkan, A.; Chattopadhyay, B.; Baert, K.; Poleunis, C.; Tromont, A.; Snyders, R.; Delcorte, A.; Terryn, H.; Delplancke-Ogletree, M. P.; Geerts, Y. H.; Reniers, F. N-Doped TiO2 Photocatalyst Coatings Synthesized by a Cold Atmospheric Plasma. *Langmuir* **2019**, *35* (22), 7161–7168. https://doi.org/10.1021/ACS.LANGMUIR.9B00784/ASSET/IMAGES/LARGE/LA-2019-00784J_0010.JPEG.

(25) Lee, C. S.; Kim, J.; Son, J. Y.; Choi, W.; Kim, H. Photocatalytic Functional Coatings of TiO2 Thin Films on Polymer Substrate by Plasma Enhanced Atomic Layer Deposition. *Appl. Catal. B Environ.* **2009**, *91* (3–4), 628–633. https://doi.org/10.1016/J.APCATB.2009.06.037.

(26) Mouele, E. S. M.; Dinu, M.; Parau, A. C.; Vladescu, A.; Myint, M. T. Z.; Kyaw, H. H.; Al-Sabahi, J.; Al-Abri, M.; Dobretsov, S.; Al Belushi, M. A.; Al-Mamari, R.; Braic, M.; Petrik, L. F. Anticorrosion Coated Stainless Steel as Durable Support for C-N-TiO2 Photo Catalyst Layer. *Mater. 2020, Vol. 13, Page 4426* **2020**, *13* (19), 4426. https://doi.org/10.3390/MA13194426.

(27) Petronella, F.; Pagliarulo, A.; Truppi, A.; Lettieri, M.; Masieri, M.; Calia, A.; Curri, M. L.; Comparelli, R. TiO2 Nanocrystal Based Coatings for the Protection of Architectural Stone: The Effect of Solvents in the Spray-Coating Application for a Self-Cleaning Surfaces. *Coatings 2018, Vol. 8, Page 356* **2018**, *8* (10), 356. https://doi.org/10.3390/COATINGS8100356.

(28) Zhai, M.; Liu, Y.; Huang, J.; Wang, Y.; Chen, K.; Fu, Y.; Li, H. Efficient Suspension Plasma Spray Fabrication of Black Titanium Dioxide Coatings with Visible Light Absorption Performances. *Ceram. Int.* **2019**, *45* (1), 930–935. https://doi.org/10.1016/J.CERAMINT.2018.09.268.
